# The Role of p53 in Determining Mitochondrial Adaptations to Endurance Training in Skeletal Muscle

**DOI:** 10.1038/s41598-018-32887-0

**Published:** 2018-10-02

**Authors:** Kaitlyn Beyfuss, Avigail T. Erlich, Matthew Triolo, David A. Hood

**Affiliations:** Muscle Health Research Centre, School of Kinesiology and Health Science, York University, Toronto, Ontario, M3J 1P3 Canada

## Abstract

p53 plays an important role in regulating mitochondrial homeostasis. However, it is unknown whether p53 is required for the physiological and mitochondrial adaptations with exercise training. Furthermore, it is also unknown whether impairments in the absence of p53 are a result of its loss in skeletal muscle, or a secondary effect due to its deletion in alternative tissues. Thus, we investigated the role of p53 in regulating mitochondria both basally, and under the influence of exercise, by subjecting C57Bl/6J whole-body (WB) and muscle-specific p53 knockout (mKO) mice to a 6-week training program. Our results confirm that p53 is important for regulating mitochondrial content and function, as well as proteins within the autophagy and apoptosis pathways. Despite an increased proportion of phosphorylated p53 (Ser^15^) in the mitochondria, p53 is not required for training-induced adaptations in exercise capacity or mitochondrial content and function. In comparing mouse models, similar directional alterations were observed in basal and exercise-induced signaling modifications in WB and mKO mice, however the magnitude of change was less pronounced in the mKO mice. Our data suggest that p53 is required for basal mitochondrial maintenance in skeletal muscle, but is not required for the adaptive responses to exercise training.

## Introduction

The tumor suppressor protein p53 is a rapid-response transcriptional regulator of numerous pathways involved in maintaining cellular homeostasis. Though extensively researched in the context of cancer and its role in the Warburg effect, few studies have examined the role of p53 in skeletal muscle, an organ that comprises 40% of total body mass^1,2^. Skeletal muscle is an exceptionally malleable tissue that can adapt to multiple physiological stimuli^1,3^, and mitochondria are the organelles that display plasticity within muscle.

Recently, the role of p53 has been examined in mitochondrial biogenesis within skeletal muscle. Exercise enhances p53 activation through kinase activation (AMPK, p38 MAPK) leading to the phosphorylation of specific p53 residues to increase its mitochondrial and nuclear localization^4–6^. Once in mitochondria, p53 functions to enhance biogenesis through its interaction with Tfam, and its requirement for the expression of mtDNA gene products in response to exercise^7–9^. Acute exercise has specifically been shown to increase p53 serine15 phosphorylation and subsequent localization to subsarcolemmal (SS) and intermyofibrillar (IMF) mitochondria, with concomitant increases in mtDNA copy number and elevated COX-I protein^5,6^. p53 additionally localizes to the nucleus where it can bind to the promoters of PGC-1α and nuclear genes encoding mitochondrial proteins (NuGEMPs) such as Tfam, COX IV, and SCO2, to upregulate transcription^5,10,11^. p53 mitochondrial localization with acute exercise seems to be preferred over nuclear accumulation^5^, however it has yet to be established whether exercise training modifies this distribution and further, and whether p53 is required within the mitochondria for adaptation purposes.

p53 is also known to play a role in regulating additional mitochondrial-dependent signaling pathways, including autophagy/mitophagy and apoptosis. Within the cytosol, p53 acts as an upstream endogenous repressor of autophagy, through its interaction with the ULK1 complex^12,13^. When nuclear localized, p53 can regulate autophagy through transcription of upstream activators AMPK and TSC2^14^, as well as lysosomal genes^6,15,16^. Furthermore, p53 monitors ubiquitination and regulates autophagy flux through LC3 and p62 modulation^6,15,17^. Although autophagic flux and lysosomal activation in response to acute exercise does not depend on p53, it is required for the regulation of ubiquitination^6^. However, it has not been established how the autophagy pathway is regulated by p53 with exercise training. p53 is also well-known for its role in regulating apoptosis, since it can transcriptionally regulate numerous pro-apoptotic genes including Bax and Bid to induce DNA fragmentation^4,18,19^. Furthermore p53 itself can localize to the mitochondrial surface where it can regulate permeability transition pore kinetics^19,20^. Chronic exercise has been previously shown to reduce the Bax:Bcl-2 ratio^21,22^ concomitant with reductions in cytochrome c and AIF protein release^23^, indicative of anti-apoptotic adaptations in mitochondria. However, the role of p53 in mediating these exercise training effects on the apoptotic pathway is still unknown. Furthermore, literature published on the role of p53 in regulating autophagy, apoptosis, mitochondrial biogenesis and metabolism, have been established largely through the use of a whole body p53 knockout model. Though this research has elucidated novel roles for p53, recently published work utilizing a muscle-specific p53 knockout model did not observe any reductions in mitochondrial content or the expression of nuclear genes encoding mitochondrial proteins^24,25^. Thus, the purpose of this study was to elucidate the role of p53 in regulating skeletal muscle adaptations to endurance training, particularly related to the mitochondrial biogenesis, autophagy, and apoptosis pathways. To accomplish this, we utilized a muscle-specific p53 deficient mouse and when relevant, compared this model to traditionally employed p53 whole body knockout animals under identical endurance training stimuli.

## Results

### Exercise-induced p53 localization to the nucleus is reduced with training and increases in the mitochondria

**Figure 1:**
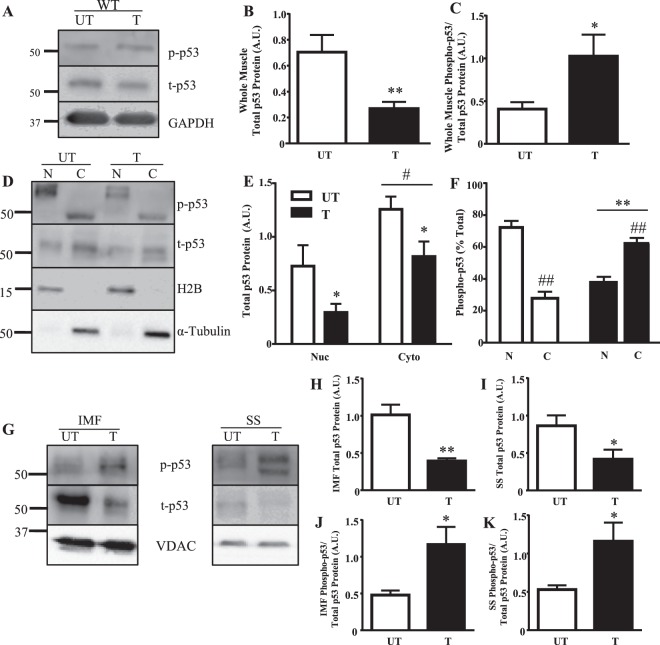
p53 subcellular localization with training. (**A**) Whole muscle (**B**) total p53 protein and (**C**) phosphorylated p53-Ser^15^ protein was measured in WT mice. GAPDH was utilized as the loading control for all immunoblotting in whole muscle (n = 6–7/group); *p ≤ 0.05, **p ≤ 0.01, UT vs. T, Student’s t-test. (**D**) p53 protein was measured in nuclear and cytosolic fractions in WT mice. H2B was used as a nuclear loading control and α-tubulin was used as a cytosolic loading control. Basally and with training, nuclear and cytosolic (**E**) total p53 protein, and (**F**) percent total of phosphorylated p53-Ser^15^ protein was measured (n = 4–5/group); *p ≤ 0.05, **p ≤ 0.01, UT vs. T; ^#^p ≤ 0.05, ^##^p < 0.01, Nuclear vs. Cytosolic (n = 4–5/group), Student’s t-test and 2-way ANOVA. A main effect of training was observed. (**G**) Mitochondrial p53 protein was examined in SS and IMF mitochondrial subfractions. VDAC was utilized as a mitochondrial loading control. Total p53 protein in (**H**) IMF, and (**I**) SS mitochondria was measured (n = 4–5/group); *p ≤ 0.05, **p ≤ 0.01, UT vs. T, Student’s t-test. Activated p53, corrected for total, was additionally measured in (**J**) IMF, and (**K**) SS mitochondria (n = 4–5/group); *p ≤ 0.05, UT vs. T, Student’s t-test. Data are presented as mean ± SEM.

p53 protein expression in whole muscle and subcellular compartments was examined following acute exercise in both trained and untrained WT mice. Total p53 protein was reduced by 2.6-fold with training (Fig. 1A,B). However, the amount of activated p53 present, measured by Ser^15^ phosphorylation, was increased by 2.5-fold in the trained state (Fig. 1A,C). p53 was largely localized to the cytosol in both trained and untrained muscle (Fig. 1D,E). However, the distribution of phosphorylated p53 (Ser^15^) differed. In untrained muscle phospho-p53 was mostly nuclear-localized (70%), whereas trained muscle exhibited only 40% of phospho-p53 in the nucleus (Fig. 1D,F). Though training reduced p53 levels within SS and IMF mitochondria by 2–3-fold, phospho-p53 increased in both subfractions by ~2.3-fold following acute exercise in trained muscle (Fig. 1G–K).

### Confirmation of muscle specific mouse model

**Figure 2:**
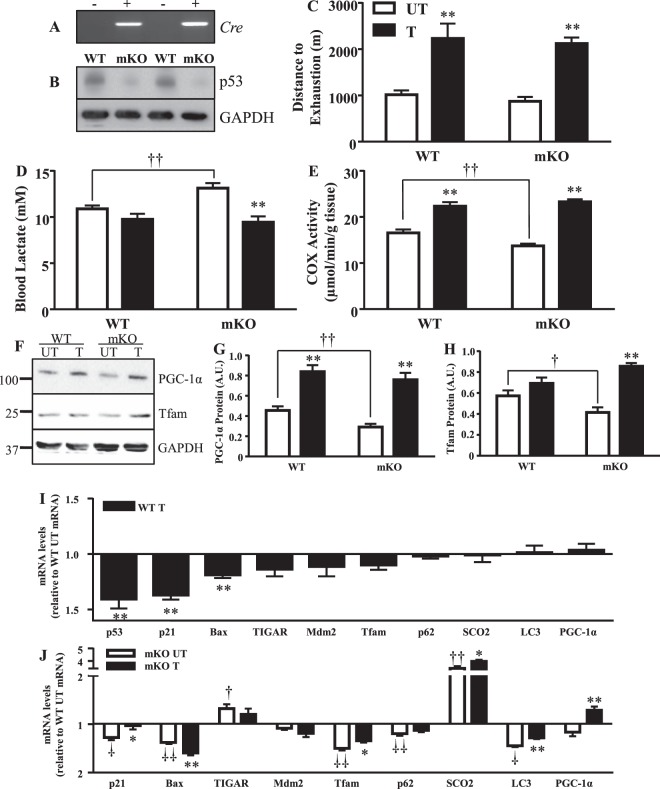
Mitochondrial content, exercise capacity, and lactate handling improves with exercise training, while gene expression is altered. The deletion of p53 in skeletal muscle of MS mKO mice was examined through (**A**) genotype against the Cre transcript, and (**B**) total p53 protein in whole muscle. Following the 6-week training/sedentary protocol, mice were subjected to an exhaustive bout of exercise to determine training-induced adaptations by measuring (**C**) distance to exhaustion (n = 6–8/group); **p ≤ 0.01, UT vs. T, 2-way ANOVA, and (**D**) final lactate production levels (n = 6–8/group), **p ≤ 0.01, UT vs. T; ^††^p ≤ 0.01, UT WT vs. mKO, Student’s t-test. Mitochondrial biogenesis with training was measured through the assessment of (**E**) COX enzyme activity, a marker of mitochondrial content (n = 6–8/group); **p ≤ 0.01, UT vs. T, 2-way ANOVA; ^††^p ≤ 0.01, UT WT vs. mKO, Student’s t-test, and (**F**) mitochondrial biogenesis markers (**G**) PGC-1α protein (n = 5–8/group); **p ≤ 0.01, UT vs. T, 2-way ANOVA; ^††^p ≤ 0.01, UT WT vs. mKO, Student’s t-test, and (**H**) Tfam protein (n = 6–7/group); **p ≤ 0.01, UT vs. T, 2-way ANOVA; ^†^p ≤ 0.05, UT WT vs. mKO, Student’s t-test. (**I**) The effect of training on mRNA transcripts of signaling pathways including cellular senescence (*p21*), apoptosis (*Bax*), metabolism (*TIGAR*), autophagy (*p62*, *LC3*), oxidative phosphorylation (*SCO2*), mitochondrial biogenesis (*PGC-1α*, *Tfam*) and *p53* and its negative regulator *Mdm2*, in WT mice. Data are presented as fold change over WT control levels (n = 8–10/group); **p ≤ 0.01, UT vs. T, Student’s t-test. (**J**) Effect of the absence of p53, and training on mRNA transcripts of signaling pathways. Data presented as fold change of mKO over WT untrained levels and as mKO trained over untrained levels (n = 7–11/group); *p ≤ 0.05, **p ≤ 0.01, mKO UT vs. T; ^†^p ≤ 0.05, ^††^p ≤ 0.01, UT WT vs. mKO, Student’s t-test. Data are presented as mean ± SEM.

The muscle-specific knockout (mKO) genotype was confirmed through the Cre recombinase transcript (Fig. 2A). In agreement with previous literature^24,25^, this successfully abolished p53 protein expression (Fig. 2B).

### Exercise training induces a leaner phenotype regardless of genotype

No differences in baseline parameters including body mass or exercise capacity were observed between genotypes (Table 1A). Training induced a leaner phenotype compared to untrained counterparts, exemplified by 28% and 56% reductions in epididymal fat mass in WT and mKO mice, respectively (Table 1B). No effect of training was observed on quadriceps or gastrocnemius skeletal muscle mass (Table 1B). Cardiac hypertrophy (7–21%), a typical consequence of training, was evident (Table 1B).

**Table 1:** Phenotypic alterations and exercise capacity under basal and exercise training conditions.

A. Pre-Training	WT		mKO	
*Initial Body wt, g*	**30**.**4** ± 0.4		**31**.**4** ± 1.0	
*Distance to Exhaustion (m)*	**1**,**286** ± 51		**1**,**156** ± 85	
**B**. **Post-Training**	**WT**		**mKO**	
	**UT**	**T**	**UT**	**T**
*Final Body wt*, *g*	**33**.**2** ± 1.1	**30**.**1** ± 0.3*	**34**.**8** ± 1.7	**27**.**8** ± 0.4*
*TA wt/body wt (mg/g)*	**2**.**0** ± 0.08	**1**.**6** ± 0.07*	**2**.**1** ± 0.08	**1**.**8** ± 0.04*
*Gastrocnemius wt/body wt*, *(mg/g)*	**6**.**3** ± 0.3	**5**.**8** ± 0.2	**6**.**3** ± 0.3	**6**.**2** ± 0.1
*Quadriceps wt/body wt (mg/g)*	**6**.**2** ± 0.1	**6**.**2** ± 0.1	**6**.**6** ± 0.2	**6**.**6** ± 0.08
*Heart wt/body wt*, *(mg/g)*	**5**.**4** ± 0.4	**5**.**8** ± 0.2	**4**.**8** ± 0.2	**5**.**8** ± 0.1*
*Epididymal Fat wt/body wt (mg/g)*	**43**.**6** ± 3.3	**31**.**5** ± 3.5*	**54**.**6** ± 3.4^†^	**23**.**9** ± 1.8*
**C**. **Mitochondrial Parameters**				
*SS Mitochondrial Yield*	**0**.**5** ± 0.07	**0**.**8** ± 0.05*	**0**.**6** ± 0.09	**0**.**8** ± 0.05*
*IMF Mitochondrial Yield*	**1**.**1** ± 0.02	**1**.**3** ± 0.1	**1**.**0** ± 0.1	**1**.**4** ± 0.08*
*SS RCR*	**3**.**8** ± 0.8	**12**.**9** ± 5.3	**4**.**6** ± 0.6	**7**.**2** ± 1.8
*IMF RCR*	**6**.**1** ± 0.2	**10**.**1** ± 2.9	**7**.**0** ± 1.1	**12**.**1** ± 4.5

(**A**) Pre-training variables (initial body mass and distance to exhaustion) were compared between genotype in the muscle specific (n = 13–23/group). (**B**) Phenotypic variables were measured following the training or sedentary program (n = 6–10/group); *p ≤ 0.05, UT vs. T; ^†^p ≤ 0.05, WT vs. mKO, 2-way ANOVA and Student’s t-test. (**C**) Mitochondrial parameters measured in SS and IMF mitochondrial subfractions (n = 5–10/group); *p ≤ 0.05, UT vs. T, 2-way ANOVA and Student’s t-test. Data are presented as mean ± SEM. Abbreviations: RCR, respiratory control ratio; TA; tibialis anterior; SS, subsarcolemmal; IMF, intermyofibrillar.

### p53 is not required for exercise capacity adaptations to a long term training program

A post-training performance test was employed to examine the necessity of p53 for improvements with training. The pre-training performance test elucidated no difference in baseline exercise capacity between genotype (Table 1A), however the mKO mice exhibited a 17% increase in blood lactate (Fig. 2D). Following the training program, both WT and mKO mice significantly improved their exercise capacity by ~2.3-fold relative to the untrained mice (Fig. 2C). Furthermore, blood lactate levels were reduced by 12–27% in both the WT and mKO trained mice, an expected adaptation to training (Fig. 2D).

### p53 is required to maintain basal mitochondrial content and function, but not for exercise training-induced adaptations

**Figure 3:**
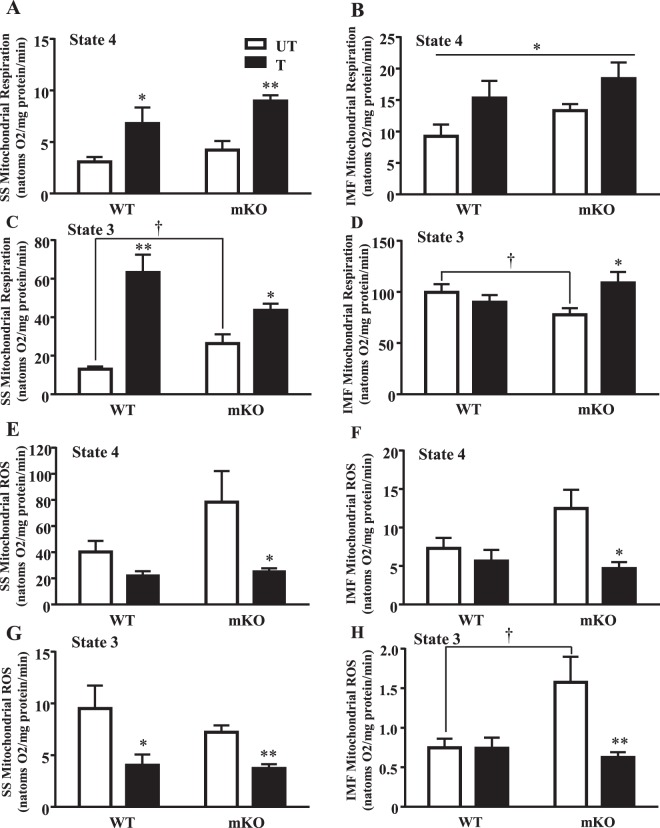
Mitochondrial respiration improves with exercise training while ROS is reduced. State 4 respiration was measured in (**A**) SS (n = 6–7/group); *p ≤ 0.05, **p ≤ 0.01, UT vs. T, Student’s t-test and 2-way ANOVA, and (**B**) IMF mitochondria (n = 6–7/group); *p ≤ 0.05, main effect of genotype and training, 2-way ANOVA. State 3 respiration was further measured in (**C**) SS (n = 6/group); *p ≤ 0.05, **p ≤ 0.01, UT vs. T, Student’s t-test and 2-way ANOVA; ^†^p ≤ 0.05, UT WT vs. mKO, Student’s t-test, and (**D**) IMF mitochondria (n = 6–7/group); *p ≤ 0.05, UT vs. T; ^†^p ≤ 0.05, UT WT vs. mKO, Student’s t-test. State 4 ROS levels were measured in (**E**) SS, and (**F**) IMF mitochondria (n = 6–8/group); *p ≤ 0.05, UT vs. T, 2-way ANOVA. State 3 ROS levels were measured in (**G**) SS, and (**H**) IMF mitochondria (n = 6–8/group); *p ≤ 0.05, **p ≤ 0.01, UT vs. T, Student’s t-test and 2-way ANOVA; ^†^p ≤ 0.05, UT WT vs. mKO, 2-way ANOVA. Data are presented as mean ± SEM.

Untrained mKO mice exhibited a 17% reduction in mitochondrial content, as measured by COX activity, compared to the untrained WT mice (Fig. 2E). In addition, PGC-1α protein was reduced by 36%, accompanied by a 28% reduction in Tfam levels (Fig. 2F–H). In response to training, both WT and mKO mice increased their mitochondrial content by 1.3- and 1.7-fold respectively, relative to untrained counterparts (Fig. 2E). SS and IMF mitochondrial yield increased with training by ~31% and ~22% respectively, with no reduction observed in the mKO mice under basal conditions (Table 1C). The respiratory control ratio measured in mitochondrial subfractions was not significantly affected by training (Table 1C). Training increased PGC-1α protein levels by 1.8–2.6-fold, regardless of genotype (p < 0.05; Fig. 2F,G). Tfam protein levels also increased in the mKO mice with training by 2.1-fold, while only a modest elevation was observed in the WT mice (Fig. 3F,H). Therefore mitochondrial biogenesis markers increase with exercise training, even in the absence of p53.

Mitochondrial function was assessed by measuring respiration and reactive oxygen species production (ROS) in both SS and IMF subfractions. No baseline differences in SS or IMF state 4 mitochondrial respiration were observed in the absence of p53 (Fig. 3A,B). However, state 3 respiration in SS mitochondria was 2-fold higher in the mKO mice, but was 22% lower in the IMF subfraction, indicating a differential dependency on p53 (Fig. 3C,D). With exercise training, state 4 respiration increased by ~2.2-fold in SS mitochondria and by 1.4–1.7-fold in IMF mitochondria in both genotypes (p < 0.05; Fig. 3A,B). State 3 respiration in SS mitochondria improved by 4.8-fold in the WT mice with training, but only increased by 1.7-fold in the mKO mice (Fig. 3C). IMF state 3 respiration did not improve in the WT mice with training, but training did attenuate the deficit observed in the mKO mice, such that state 3 respiration was similar to control values (Fig. 3D).

In the absence of p53, state 3 and 4 ROS levels were elevated under basal conditions in both the SS and IMF subfractions (Fig. 3E,F,H). Exercise training attenuated state 3 and 4 SS ROS levels by 23–58% in WT mice and normalized ROS emission in both SS and IMF subfractions under state 3 and state 4 conditions in mKO mice (p < 0.05; Fig. 3E–H). Therefore, the presence of p53 is not required for the adaptive decreases in mitochondrial ROS emission with training. Concomitant with these changes, training induced similar decreases in the anti-oxidant protein Nrf2 as well as its regulator Keap 1 in both WT and mKO mice (Fig. S2).

### Transcripts of genes related to downstream p53 signaling targets alter with exercise training

The mRNA transcripts of signaling pathways regulated by p53 were examined. Training downregulated *p53* (1.4-fold), *p21* (~1.4-fold), and *Bax* (1.2-fold) transcripts (p < 0.05; Fig. 2I). *TIGAR*, *Mdm2*, *Tfam*, *LC3*, *p62*, *SCO2*, and *PGC-1α* mRNA transcripts were not significantly altered with training. In the absence of p53 under basal conditions, there was a 1.2–1.6-fold reduction in the transcripts of genes involved in mitochondrial biogenesis (*Tfam*), autophagy (*LC3*, *p62*), and cell cycle arrest/cell death (*p21*, *Bax*) signaling pathways (p < 0.05; Fig. 2J). In contrast, *TIGAR* and *SCO2* mRNA levels increased in the absence of p53. The effect of training was evaluated to determine if exercise could reverse the gene expression pattern defined by the absence of p53. With training, the decrease in *PGC-1α* mRNA was reversed, exhibited by a ~1.3-fold increase above WT untrained levels. The decrease in *Bax* mRNA and the increase in *SCO2* were further amplified with training (p < 0.05) while the reductions in *p21*, *Tfam*, and *LC3* transcripts were normalized. Changes in the transcripts of *TIGAR* and *p62*, brought about by p53 deficiency, did not respond to training, indicating a strong dependence on p53 for expression. *Mdm2* was not significantly affected by either training, or the absence of p53, at the transcript level.

### Differential apoptotic release occurs in mitochondrial subfractions however, training reduces intrinsic mitochondrial apoptosis

**Figure 4:**
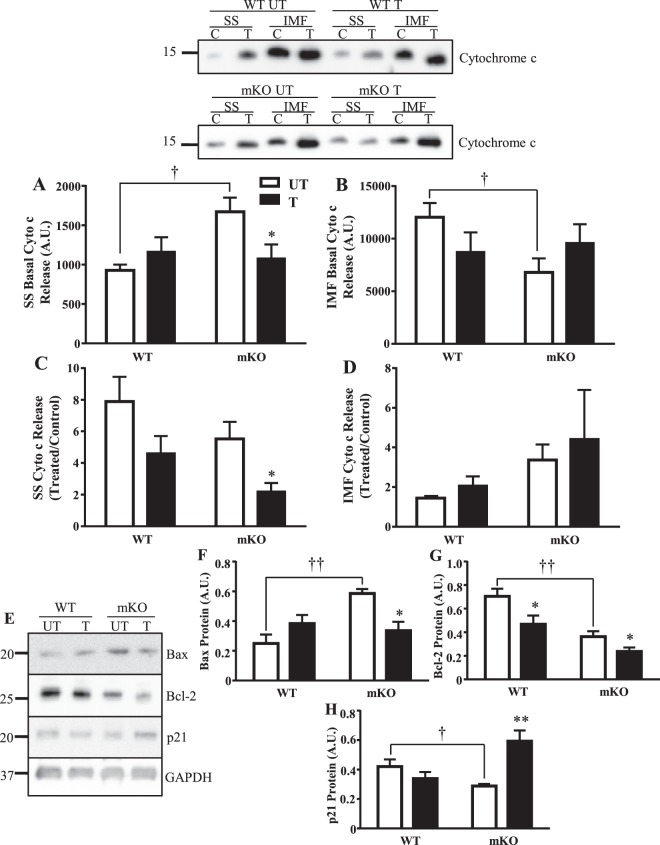
Effect of training and p53 on mitochondrially-mediated apoptosis. In the absence of p53 basally, (**A**) greater cytochrome c release is observed in SS mitochondria with training reducing release (n = 5–8/group); *p ≤ 0.05, UT vs. T, One-way ANOVA; ^†^p ≤ 0.05, UT WT vs. mKO, Student’s t-test, and (**B**) reduced basal release is observed in IMF mitochondria (n = 7–14/group); ^†^p ≤ 0.05, UT WT vs. mKO, Student’s t-test. Under apoptotic stimuli (H_2_O_2_), cytochrome c release was reduced with training in **C**) SS mitochondria (n = 6–10/group); *p ≤ 0.05, UT vs. T, One-way ANOVA, and (**D**) did not change in IMF mitochondria (n = 6–12/group). (**E**) Apoptosis, anti-apoptosis, and cellular senescent proteins were measured in whole muscle; (**F**) Bax protein (n = 4–6/group); *p ≤ 0.05; UT vs. T; ^††^p ≤ 0.01, UT WT vs. mKO, 2-way ANOVA; (**G**) Bcl-2 protein (n = 5–8/group); *p ≤ 0.05, UT vs. T, Student’s t-test; ^††^p ≤ 0.01, UT WT vs. mKO, 2-way ANOVA; (**H**) p21 cellular senescent protein (n = 5–6/group); **p ≤ 0.01, UT vs. T, 2-way ANOVA; ^†^p ≤ 0.05, UT WT vs. mKO, Student’s t-test. Data are presented as mean ± SEM.

To assess mitochondrial apoptotic susceptibility, we measured cytochrome c protein release from isolated organelles in the presence (H_2_O_2)_ and absence of apoptotic stimuli. Under basal conditions, cytochrome c release from SS mitochondria was increased by 81% in the absence of p53, but was reduced by 44% in the IMF subfraction compared to WT counterparts (Fig. 4A,B). Training attenuated the elevated cytochrome c release rate in the SS subfraction of mKO mice to reach WT levels (p < 0.05; Fig. 4A). An attenuation of cytochrome c release from SS mitochondria by 41–60% in the WT and mKO mice was also observed in the presence of H_2_O_2_ (Fig. 4C). There was no effect of training or genotype on H_2_O_2_–induced cytochrome c release in the IMF subfraction (Fig. 4D).

To relate patterns of cytochrome c release to upstream activators, we measured p21, Bax, and Bcl-2 protein in whole muscle lysates. In the absence of p53, Bax protein increased by 2.3-fold, whereas Bcl-2 and p21 proteins were reduced by 49% and 31%, respectively (Fig. 4E–H). Training reduced Bax protein by 43% in the mKO mice, to values reaching WT control levels (Fig. 4E,F). Bcl-2 protein was reduced in both genotypes by ~34% with training (Fig. 4E,G). Though p21 was unaffected by training in the WT mice, training induced a significant augmentation (2.1-fold) in p21 expression in the mKO mice (Fig. 4E,H). Therefore, p53 does not appear to be required for the anti-apoptotic adaptations induced by exercise training.

### Exercise training increases autophagy signaling

**Figure 5:**
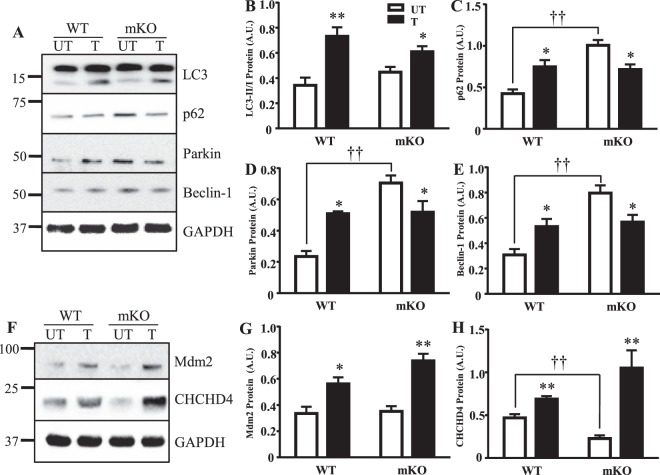
Autophagy protein response and regulators of p53 expression in the absence of p53 and with training. (**A**) Proteins in the autophagy pathway were measured; (**B**) LC3-II/LC3-I ratio (n = 6–8/group); *p ≤ 0.05, **p ≤ 0.01, UT vs. T, 2-way ANOVA, (**C**) p62 (n = 5–8/group), *p ≤ 0.05, UT vs. T; ^††^p ≤ 0.01, UT WT vs. mKO, 2-way ANOVA, (**D**) Parkin (n = 5–7/group); *p ≤ 0.05, UT vs. T, Student’s t-test and 2-way ANOVA; ^††^p ≤ 0.01, UT WT vs. mKO, 2-way ANOVA, and (**E**) Beclin-1 (n = 5–7/group); *p ≤ 0.05, UT vs. T, Student’s t-test; ^††^p ≤ 0.01, UT WT vs. mKO, 2-way ANOVA. (**F**) Regulators of p53 expression (Mdm2) and its mitochondrial localization (CHCHD4) were next examined. Whole muscle protein expression of (**G**) Mdm2 (n = 5–6/group); *p ≤ 0.05, **p ≤ 0.01, UT vs. T, 2-way ANOVA, and (**H**) CHCHD4 (n = 5–6/group); **p ≤ 0.01, UT vs. T, Student’s t-test and 2-way ANOVA; ^††^p ≤ 0.01, UT WT vs. mKO, Student’s t-test, was measured. Data are presented as mean ± SEM.

We evaluated the potential role of p53 in mediating autophagy through the examination of LC3, p62, Parkin and Beclin-1. In the absence of p53 under basal conditions, the autophagy proteins p62, Parkin and Beclin-1 were upregulated by 2.4–3-fold (Fig. 5A,C–E). LC3-I and LC3-II levels were unaffected (Fig. 5A,B). It should be noted that this pattern differs somewhat from our previous results in whole-body KO animals^26^, possibly as a result of the model, or the fact that all animals were subjected to acute exercise in the current study. Training induced relatively similar 1.7–2.2-fold increases in all of these autophagy proteins in WT mice (Fig. 5A–E). This increase was attenuated for LC3-II, and reversed for p62, Parkin and Beclin-1 in the absence of p53, suggesting that training can normalize the aberrant expression of these proteins in p53 null muscle.

### Targeted regulation of p53 protein

The effect of training was examined on proteins that determine p53 steady state levels (Mdm2) and mitochondrial localization (CHCHD4). Mdm2 levels were unaffected by the absence of p53, and were increased by 1.7–2.1-fold with training (Fig. 5F,G). CHCHD4 protein was reduced by 52% in the absence of p53 under basal conditions (p < 0.05; Fig. 5F,H). Training induced modest (1.5-fold) and large (4.6-fold) increases in CHCHD4 expression in WT and mKO mice, respectively.

### Signaling pathways and mitochondrial properties in muscle-specific (mKO) and whole body (WB) p53 deletion models

**Figure 6:**
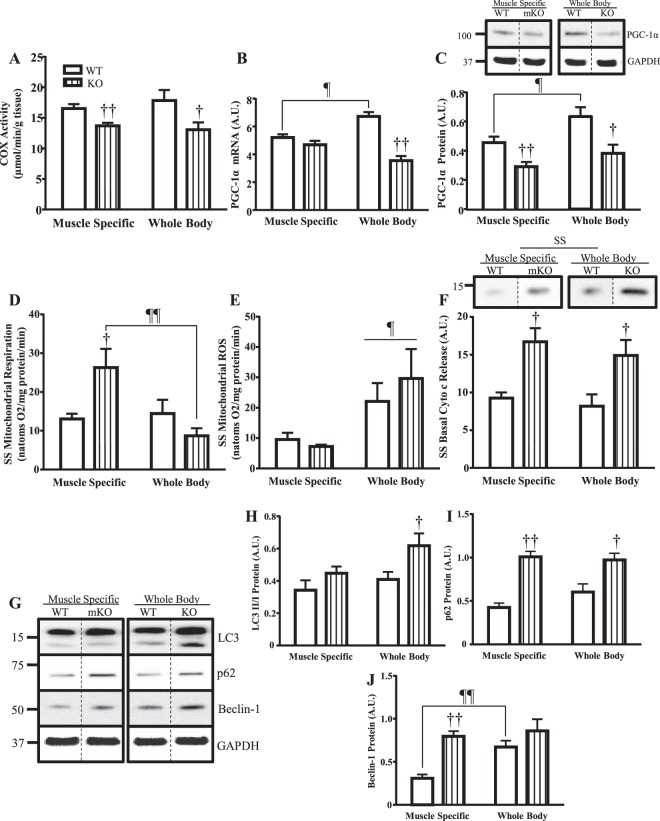
Basal mitochondrial biogenesis, apoptosis, and autophagy signaling comparisons between mouse models. Muscle specific (mKO) and whole body (WB) p53 deletion models were compared under basal conditions. Mitochondrial biogenesis was measured through (**A**) COX enzyme activity (n = 6–8/group); ^†^p ≤ 0.05, ^††^p ≤ 0.01, UT WT vs. KO, Student’s t-test and 2-way ANOVA, and whole muscle (**B**) PGC-1α mRNA (n = 5–10/group); ^††^p ≤ 0.01, UT WT vs. KO, 2; ^¶^p ≤ 0.05, WT MS vs. WB, 2-way ANOVA, and (**C**) PGC-1α protein (n = 5–7/group); ^†^p ≤ 0.05, ^††^p ≤ 0.01, UT WT vs. KO, Student’s t-test and 2-way ANOVA; ^¶^p ≤ 0.05, WT MS vs. WB, Student’s t-test. Immunoblots were retrieved from the same blot but were spliced for direct comparison of untrained animals. Full-length blot is presented in Supplementary Figure 2. SS mitochondrial state 3 (**D**) respiration (n = 6/group); ^†^p ≤ 0.05, UT WT vs. KO, Student’s t-test; ^¶¶^p ≤ 0.01, KO MS vs. WB, 2-way ANOVA, and (**E**) ROS emission (n = 6–7/group); ^¶^p ≤ 0.05, MS vs. WB, Student’s t-test and 2-way ANOVA, was measured. (**F**) Basal cytochrome c release in SS mitochondria (n = 5–9/group); ^†^p ≤ 0.05, UT WT vs. KO, Student’s t-test and 2-way ANOVA. Immunoblots were spliced for direct comparison of untrained animals. Full-length blot is presented in Supplementary Figure 3. (**G**) Autophagy was further examined through whole-muscle analysis of autophagy proteins including (**H**) LC3-II/LC3-I ratio (n = 6–8/group); ^†^p ≤ 0.05, UT WT vs. KO, Student’s t-test, (**I**) p62 (n = 5–8/group); ^†^p ≤ 0.05, ^††^p ≤ 0.01, UT WT vs. KO, 2-way ANOVA, and (**J**) Beclin-1 (n = 5–7/group); ^††^p ≤ 0.01, UT WT vs. KO, 2-way ANOVA; ^¶¶^p ≤ 0.01, WT MS vs. WB, Student’s t-test. Immunoblots were retrieved from the same blot but were spliced for direct comparison of untrained animals. Full-length blot is presented in Supplementary Figure 4.Data are presented as mean ± SEM.

A limited comparison of mitochondrial parameters between WB p53 KO and mKO models under basal conditions was performed. Mitochondrial content in the mKO and WB KO mice was reduced by 17% and 27% respectively, with no difference in levels between models (Fig. 6A). PGC-1α mRNA was not altered in the mKO mice, however it was reduced by 47% in WB KO mice compared to WT counterparts (p < 0.05; Fig. 6B). Furthermore, PGC-1α mRNA and protein was higher in the WT mice of the WB model (Fig. 6B,C). PGC-1α protein was reduced by 36% and 40% in the mKO and WB KO mice, respectively (Fig. 6C. Full blot shown in Fig. S3). Differences in state 3 respiration were observed in the absence of p53 in SS mitochondria whereby mKO mice had 2-fold higher respiration, and WB KO mice had a 1.4-fold lower state 3 respiration compared to WT counterparts (Fig. 6D). State 3 ROS in SS mitochondria was not elevated in the absence of p53 in either model, however the WB mice displayed greater (2.5–4.1-fold) ROS levels overall (Fig. 6E). Basal cytochrome c release in SS mitochondria was elevated by ~80% in the absence of p53 in both models compared to WT mice (Fig. 6F. Full blot shown in Fig. S4).

The autophagy signaling pathway was measured through the examination of LC3, p62, and Beclin-1 proteins. Though LC3-I remained relatively constant between genotypes, activated LC3-II protein was increased in the WB KO mice, leading to an elevation in the LC3 II/I ratio (p < 0.05; Fig. 6G,H. Full blot shown in Fig. S5). p62 protein was elevated by 2.4- and 1.6-fold in the mKO and WB KO mice respectively (Fig. 6G,I). Beclin-1 protein was increased in the mKO mice by 2.6-fold, but the higher level evident in the WB WT control mice precluded a significant increase in the KO animals (Fig. 6G,J). Additional experiments were conducted to illustrate potential differences in WB and mKO mice. With regard to endurance and strength, no differences were observed following a battery of tests (Fig. S6). In addition, IMF mitochondria exhibited a decrease in cytochrome c release in both the mKO and WB mice in the absence of p53 (Fig. S7A), indicative of reduced mitochondrially-mediated apoptosis. Despite this, the overall level of DNA fragmentation was greater in WB mice, compared to mKO counterparts (Fig. S7B; n = 6–8/group). This may be a result of the differential expression of pro- and antiapoptotic proteins observed between the WB and mKO mice (Fig. S8).

### Understanding the combined effect of genotype, training, and mouse model on exercise capacity adaptations and mitochondrial biogenesis

**Figure 7:**
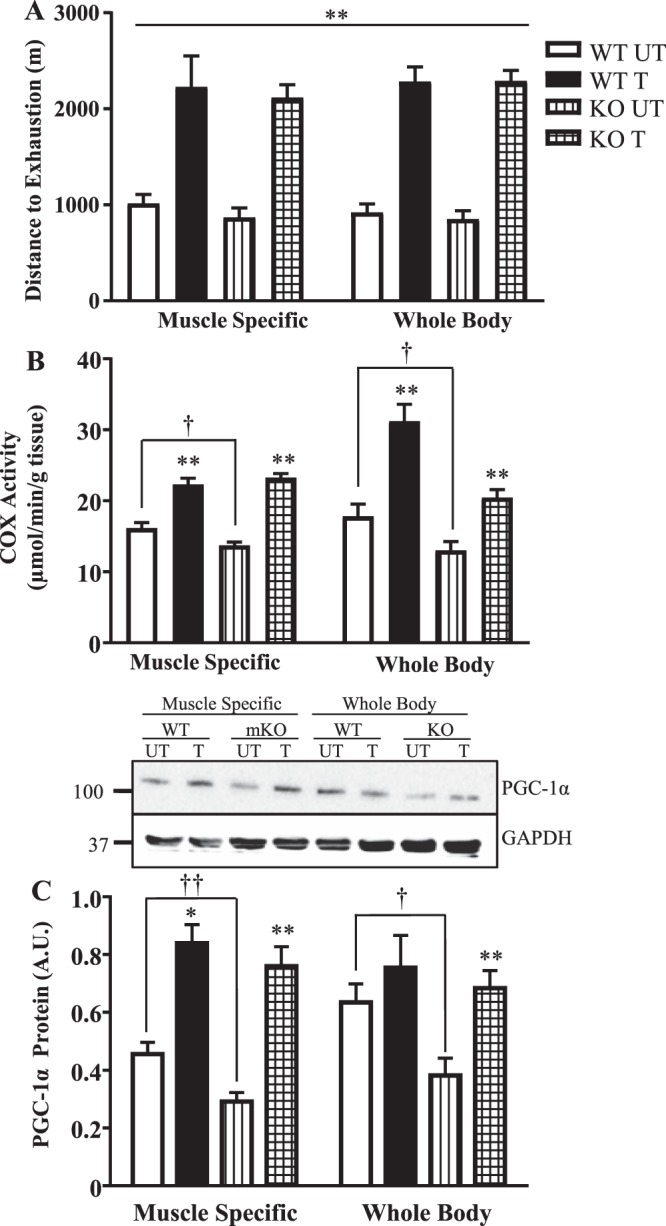
Adaptive mitochondrial responses with training in two mouse models. Following the 6-week training/sedentary protocol, the WT and muscle-specific (mKO) and whole-body (WB) p53 knockout mice were subjected to an exhaustive exercise test. (**A**) Distance to exhaustion in both mouse models (n = 6–9/group); **p ≤ 0.01, UT vs. T, 2-way ANOVA. (**B**) COX activity (n = 6–8/group); **p ≤ 0.01, UT vs. T, Student’s t-test and 2-way ANOVA; ^†^p ≤ 0.05, UT WT vs. KO, Student’s t-test. **C**) PGC-1α protein (n = 5–10/group); *p ≤ 0.05, **p ≤ 0.01, UT vs. T, Student’s t-test and 2-way ANOVA; ^†^p ≤ 0.05, ^††^p ≤ 0.01, UT WT vs. KO, Student’s t-test. Some of the data presented in Fig. 2 are replicated here in Fig. 7 for comparison. Data are presented as mean ± SEM.

The adaptive responses to training were measured in both mouse models. Exercise capacity was significantly improved (~2.5-fold) following the training program in all mice regardless of genotype or model (Fig. 7A). Training increased mitochondrial content by 35–70% in the MS mice, attaining similar levels between genotypes post-training (Fig. 7B). In the WB mice, training increased mitochondrial content by 75% in the WT mice, and by 57% in the KO mice. In the absence of p53, training elevated PGC-1α protein levels to a similar extent in both WB KO and mKO mice, thus attenuating the deficit induced by p53. Therefore, the absence of p53 does not impact the improvements in performance, mitochondrial content, or expression of PGC-1α in response to training.

## Discussion

The function of p53 in the transcriptional regulation of numerous signaling pathways is well established. However, its role in maintaining mitochondrial content and function, as well as the adaptive responses to endurance training remains unresolved. Thus, we sought to comprehensively evaluate this function of p53 by using muscle-specific p53 knockout animals. Our results reveal that 1) training reduces p53 at the mRNA and protein levels, but increases the proportion of activated p53, particularly in mitochondria, 2) p53 is important for regulating basal mitochondrial biogenesis, respiration, ROS emission and apoptosis, but is not essential for training-induced adaptations in mitochondrial content, exercise capacity, and lactate handling, 3) p53 is not required for autophagy signaling but may be necessary for substrate clearance^6,15^, a process improved with training, 4) p53 is important for maintaining the expression of specific transcripts regulating mitochondrial biogenesis, metabolism, autophagy, apoptosis and cellular senescence, both basally and with training, and 5) training can improve mitochondrial content and function, as well as attenuate the deficits in mitochondrial function induced by the absence of p53.

It is well-established that acute exercise results in p53 re-localization. We and others have shown that p53 can be redirected to the mitochondria^5,27^ and nucleus^28–30^ with acute exercise, concomitant with the activation of both AMPK and p38 MAPK kinases^5,31,32^ and the phosphorylation of p53 on Ser^5,15,33^. This is important for the transcriptional activation of nuclear and mitochondrial gene products, as well as for the dissociation of p53 from its negative regulator Mdm2^5,9,34–36^.The results presented in our study extend these findings to show that endurance training modifies this subcellular redistribution during acute exercise. Though nuclear accumulation of p53 does occur with acute exercise^5,28–30,37,38^, training resulted in the re-localization of activated p53 out of the nucleus, leading to its cytosolic accumulation (Fig. 1F) where it can then localize to mitochondria (Fig. 1J,K). p53 mitochondrial accumulation was likely facilitated by enhanced CHCHD4 levels (Fig. 5H), a component of the mitochondrial import machinery, which specifically targets p53 to increase trafficking into the organelle^30^. Once inside, p53 can maintain mitochondrial genome integrity by interacting with Tfam, increasing mtDNA transcription, and enhancing polymerase-γ activity^5,7,8,26,39^.

We sought to identify the mitochondrial impairments in muscle consequent to the absence of p53, and the potential corrective effects of a chronic endurance training program. Under basal conditions, the presence of a genome-wide p53 deletion has been shown to result in morphological disruptions of SS and IMF mitochondria, reduced mitochondrial content and mtDNA copy number, and decreases in numerous transcriptional targets including PGC-1α and Tfam^4,5,10,26,40^. Functional deficits include impaired state 3 respiration and elevated ROS levels in IMF mitochondria, altered DNA fragmentation and enhanced apoptotic potential, evidenced through increased cytochrome c release from SS mitochondria^4,41,42^. Our data reveal that the muscle-specific absence of p53 results in a modest, but significant reduction in mitochondrial content, along with decreases in both Tfam and PGC-1α mRNA and protein. Functional mitochondrial deficits were also observed, including impaired state 3 respiration, elevated ROS levels and increased pro-apoptotic signaling, accompanied by an increase in the Bax:Bcl-2 protein ratio. Interestingly, a recent study employing 8-week old muscle-specific p53 KO mice^24^ observed no deficits in mitochondrial proteins or mRNA. This difference in results may be developmentally-related, as our study was conducted using ~3 month old mice for comparison. This would suggest that the influence of p53 on mitochondrial function is dependent on age. Although cage activity, based on voluntary running wheel data, differs markedly between whole body p53 knockout and WT animals^4^, we found no differences in voluntary wheel running activity over a 2-week period between mKO and WT mice (n = 3–4). Thus, differences in activity level do not appear to be the cause of the reduced mitochondrial content in mKO mice.

We additionally probed the autophagy pathway, known to be altered in the absence of p53^4,6,12,13,15,43,44^. Elevations in p62, Parkin, and Beclin-1 autophagy proteins were observed in the absence of p53, with a trend for an increase in the LC3 II/I ratio. These data suggest that autophagic signaling is enhanced in the absence of p53, but that clearance of autophagosomes, and substrates such as p62, is impaired. Similar results have previously been shown^6^, whereby the absence of p53 led to elevated p62 protein and reduced ubiquitination. This impaired clearance could contribute to the accumulation of dysfunctional mitochondria that we observed in the mKO mice (Fig. 3D,H).

We also examined the consequences of muscle-specific p53 deletion on muscle mass and physiological performance. Analogous to previous work^25^, our mKO mice revealed no baseline differences in body or hindlimb mass, indicating that p53 is not required for the maintenance of skeletal muscle mass. While one study identified significant reductions in whole body exercise performance and aerobic capacity, evidenced by 3-fold higher lactate levels and impaired aerobic swimming performance as a result of p53 deletion^4,10^, another investigation revealed no effect on performance during an acute bout of exhaustive exercise^5^. We confirmed these findings, whereby our mKO mice displayed similar pre-training exercise performance as the WT mice. However, the lower oxidative capacity of these mice resulted in higher blood lactate following their exercise test, suggesting a greater reliance on glycolysis to achieve this same performance.

We sought to determine whether p53 is necessary for adaptations to exercise training. In contrast to a previous study using a whole body p53 knockout model in which a reduced adaptive capacity was noted^10^, we observed that WT and mKO mice respond with similar phenotypic and physiological adaptations to training, as shown by >2-fold improvement in exercise capacity and an attenuation in final lactate levels. Mitochondrial content was increased in the mKO mice by 70%, to attain similar levels as the WT trained mice, which increased their COX activity by 35%. In the absence of p53, we observed similar elevations in PGC-1α and Tfam proteins. Furthermore, mitochondrial respiration was increased while ROS emission was reduced in the mKO mice, indicating that training improved the mitochondrial deficits induced basally by the absence of p53. Apoptosis was down-regulated with exercise training, as evidenced by reduced cytochrome c release from SS mitochondria and attenuated Bax protein levels in the mKO mice. Proteins of the autophagy pathway, including Parkin, Beclin-1, and LC3-II were significantly upregulated in the WT mice with training, as shown in other models of chronic exercise^45–47^. In contrast, training reduced the already elevated levels of these proteins in mKO mice toward levels observed in the WT trained mice. Of particular relevance is the training-induced reduction in p62 protein, in the absence of a change in p62 mRNA, signifying enhanced p62 degradation via autophagic flux. Thus, training induces the activation of redundant signals to increase mitochondrial biogenesis and autophagy, and reduce apoptosis, to compensate for the loss of p53, resulting in similar final adaptations between groups.

As most literature on the role of p53 in skeletal muscle has been performed using a whole body (WB) p53 deletion model, we inquired as to whether deficits observed in the WB model are a result of the loss of p53 in muscle, or a consequence of its absence in alternative tissues. Though we observed similar directional alterations in mitochondrial content, autophagy proteins, PGC-1α mRNA and protein, state 3 respiration and ROS emission in SS mitochondria of both WB p53 KO and mKO models basally, the magnitude of change was less pronounced in the mKO mice^4–6,10^. This may be a result of the lingering, yet vanishingly low, p53 protein in the mKO mice, or a consequence of blood-borne metabolic, endocrine, or immunological influences on muscle as a result of a genome-wide p53 deletion. Whatever the cause, it is evident that chronic exercise is able to combat these defects to produce similar adaptations in performance, mitochondrial function, and PGC-1α protein between these two experimental models.

This study has sought to provide a greater understanding of the role of p53 in mediating exercise-induced adaptations in muscle. Though p53 is required for mitochondrial maintenance and function, autophagy and apoptotic regulation, its absence in muscle does not cause an impairment in endurance exercise performance. Thus, redundant signaling exists to compensate for its loss, allowing for beneficial exercise-induced mitochondrial adaptations to occur, thereby improving muscle health. Our data also indicate that exercise, in the form of a progressive and regulated endurance training program, can be a viable therapeutic option for individuals with mutated or non-functional p53.

## Methods

### Animal Breeding

The C57Bl/6J whole body (WB) p53 wildtype (WT) and knockout (KO) mice are from the Jackson Laboratory (California, USA). The C57Bl/6J muscle specific (MS) p53 WT and KO mice were generously provided by Dr. C.M. Adams (Iowa, USA). All procedures and protocols employing animals in this study were approved by the York University Animal Care Committee, as per protocol 2014-4. Mice were bred and treated experimentally in accordance with the animal handling and welfare ethics guidelines issued by Canadian Council on Animal Care. Details on genotyping are found in the supplemental section.

### Experimental Design

At approximately12–14 weeks of age, male C57Bl/6J WT and KO mice in the WB and MS mouse models were matched for sex and body weight. All mice were acclimatized to the treadmill prior to the first graded exercise performance test. Animals underwent the first exhaustive performance test whereby exhaustion was defined as the point when mice remained on an electric shock pad for 10 seconds despite prodding with air currents. Lactate measurements were obtained following removal from the treadmill to ensure that exhaustion was reached. Mice in both models were then randomized to a sedentary or training group. The sedentary group involved no treadmill exercise for 6 weeks, while the training group participated in a 6-week training protocol. Following 6 weeks, both the sedentary and trained groups underwent a second exercise performance test following the same parameters as the first. Approximately 48 hours later, all mice underwent an acute bout of treadmill exercise. Full details are in the supplemental methods and Fig. S1. All mice were sacrificed by cervical dislocation immediately following the acute bout for instantaneous tissue removal.

### Tissue Extraction and Experimental Organization

All mice underwent the same tissue extraction protocol. One gastrocnemius (~170 mg) and one tibialis anterior (TA) (~50 mg) were extracted and immediately snap frozen and stored at −80 °C for mRNA analysis, whole muscle western blotting, and COX enzyme activity. Part of one TA muscle (~ 30 mg) was placed in buffer and utilized for nuclear/cytosolic fractionation. The rest of the skeletal muscle (one gastrocnemius, two quadriceps femoris, two triceps) (~1000 mg) was utilized for mitochondrial fractionation and subsequent functional testing. The heart and epididymal fat were removed, weighed, and frozen in liquid nitrogen for later use. Frozen skeletal muscle samples were pulverized into a powder and cooled to the temperature of liquid nitrogen for subsequent analysis.

### Protein Extraction

Tissue powders were diluted 5X with an extraction buffer containing protease and phosphatase inhibitors. Diluted samples were rotated end-over-end at 4 °C for one hour, followed by sonication at 3 × 3 at 30% of maximum power. Samples were then centrifuged at 4 °C for 10 minutes at 16,000 g and the supernate was stored at −80 °C until required. Protein concentration was determined through the Bradford method (supplemental methods).

### Mitochondrial Fractionation

Briefly, approximately 1000 mg of fresh skeletal muscle was minced, homogenized, and subjected to differential centrifugation to yield the SS and IMF subfractions^48^. The mitochondria were re-suspended in a small volume of resuspension buffer (100 mM KCl, 10 mM MOPS, and 0.2% BSA at pH 7.4). All centrifugation steps were carried out at 4 °C. Mitochondrial homogenates were analyzed for protein content using the Bradford assay, and used immediately for mitochondrial respiration, ROS analysis, and the protein release assay before being frozen at −80 °C for later biochemical analysis via immunoblotting. Mitochondrial respiration, ROS emission, and the cytochrome c protein release assay were analyzed. Experimental details for these procedures are found in the supplemental methods.

### Nuclear and Cytosolic Fractionation

Nuclear and cytosolic fractions were prepared from freshly isolated skeletal muscle using a commercially available nuclear extraction kit (Pierce NE-PER, Rockford, IL, USA). Approximately 50 mg of skeletal muscle was minced and homogenized in CER-I buffer containing protease inhibitor cocktail Complete, EDTA free (Roche Applied Sciences, Manheim, Germany). After a series of wash steps, nuclear proteins were extracted in high salt NER buffer supplemented with protease-inhibitors. The cytosolic fraction was spun at 100,000 x g at 4 °C for 60 min to obtain a pure cytosolic fraction. Calculations were performed to assess the percentage of p53 protein within the nuclear (Nuclear p53 = p53N/p53C + p53N x 100) and cytosolic (Cytosolic p53 = p53C/p53C + p53N x 100) fractions (N = Nuclear, C = Cytosolic).

### Cytochrome c Oxidase (COX) Enzyme Activity

Mitochondrial extracts from skeletal muscle were added to a test solution containing fully reduced cytochrome c. Enzyme activity was determined as the maximal rate of oxidation of fully reduced cytochrome c measured by the change in absorbance at 550 nm in a Synergy HT microplate reader at 30 °C. The full protocol has been previously described^49^.

### Immunoblotting

Whole muscle and isolated subfractions including mitochondrial, nuclear, and cytosolic protein extracts were separated on a 10−15% sodium dodecyl sulfate polyacrylamide gel through electrophoresis (SDS-PAGE) at 120 V for ~90 minutes. Proteins were then transferred onto a nitrocellulose membrane. The membrane was stained with Ponceau Red, cut at the appropriate molecular weights, and blocked in 5% skim milk for one hour to prevent non-specific binding. The membrane strips were immunoblotted overnight at 4 °C with a primary antibody, as detailed in Table S1. Membranes were washed three times (5 min each) with tris-buffered-saline-tween-20 (TBST) solution containing 25 mM Tris-HCL (pH 7.5), 1 mM NaCl, and 0.1% Tween 20. Membranes were incubated with the appropriate secondary antibody coupled to horseradish peroxidase at room temperature for one hour. Loading controls were utilized for specific extracts. Following incubation, membranes were washed three times again in TBST, developed using an enhanced chemiluminescence (ECL) kit and Imager technology, and quantified via densitometric analysis based on signal intensity using the Sigma Scan Pro Version 5 software (Jandel Scientific, San Rafael, CA).

### RNA Isolation

Total RNA was isolated from approximately 70–80 mg of frozen powdered muscle tissue. Briefly, tissue was added to TRIzol ® reagent, homogenized, and mixed with choloroform. Samples were centrifuged at 4 °C at 16,000 g for 15 min and the aqueous supernate was transferred to a new tube with the addition of isopropanol and left overnight at −20 °C to precipitate. Samples were again centrifuged at 16,000 g for 10 min and the resultant supernate was discarded. The pellet was resuspended in 10–20 µl of sterile H_2_O. RNA concentration and quality were measured using the Nanodrop 2000 (Thermo Scientific, Wilmington, DE, USA). SuperScript ® III reverse transcriptase (Invitrogen, Carlsbad, CA, USA) was used to reverse transcribe 1.5 µg of total RNA into cDNA. The mRNA expression of *SCO2*, *TIGAR*, *Mdm2*, *p62*, *LC3*, *p52*, *p21*, *Bax*, *PGC-1α* and *Tfam*, and housekeeping genes *GAPDH* and *B2M*, were quantified using the 7500 Real-Time PCR system (Applied Biosystems Inc., Foster City, CA, USA) and SYBR® Green chemistry (PerfeC_T_a SYBR® Green Supermix, ROX, Quanta BioSciences, Gaithersburg, MD, USA). Full details for experimental analysis can be found in the supplemental methods and Table S2.

### Statistical analysis

Data were analyzed using the GraphPad Prism 7.0 software and values are reported as means ± SEM. All data were analyzed using a two-way ANOVA and Bonferroni post-tests unless otherwise indicated. Significance levels were set at p ≤ 0.05.

## Electronic supplementary material

Supplementary Information

## Data Availability

The data sets generated during and analyzed during the current study are available from the corresponding author on request.

## References

[1] Hood DA, Irrcher I, Ljubicic V, Joseph A (2006). Coordination of metabolic plasticity in skeletal muscle. J. Exp. Biol..

[2] Levine AJ, Oren M (2009). The first 30 years of p53: growing every more complex. Nat. Rev. Cancer.

[3] Adhihetty PJ, Irrcher I, Joseph AM, Ljubicic V, Hood DA (2003). Plasticity of skeletal muscle mitochondria in response to contractile activity. Exp. Physiol..

[4] Saleem A, Adhihetty PJ, Hood DA (2009). Role of p53 in mitochondrial biogenesis and apoptosis in skeletal muscle. Physiol. Genomics.

[5] Saleem A, Hood DA (2013). Acute exercise induces tumour suppressor protein p53 translocation to the mitochondria and promotes a p53-Tfam-mitochondrial DNA complex in skeletal muscle. J. Physiol..

[6] Saleem A, Carter HN, Hood DA (2014). p53 is necessary for the adaptive changes in cellular milieu subsequent to an acute bout of endurance exercise. AJP Cell. Physiol..

[7] Achanta G (2005). Novel role of p53 in maintaining mitochondrial genetic stability through interaction with DNA Pol γ. EMBO J..

[8] Yoshida Y (2003). p53 physically interacts with mitochondrial transcription factor A and differentially regulates binding to damaged DNA. Cancer Res..

[9] Saleem A, Carter H, Iqbal S, Hood DA (2011). Role of p53 within the regulatory network controlling muscle mitochondrial biogenesis. Exerc. Sport Sci. Rev..

[10] Park JY (2009). p53 improves aerobic exercise capacity and augments skeletal muscle mitochondrial DNA content. Circ. Res..

[11] Ljubicic V (2009). Molecular basis for an attenuated mitochondrial adaptive plasticity in aged skeletal muscle. Aging (Albany, NY).

[12] Tasdemir E (2008). Regulation of autophagy by cytoplasmic p53. Nat. Cell Biol..

[13] Marino G, Niso-Santano M, Baehrecke EH, Kroemer G (2014). Self-consumption: the interplay of autophagy and apoptosis. Nat. Rev. Mol. Cell Biol..

[14] Feng Z (2007). The regulation of AMPK β1, TSC2, and PTEN expression by p53: Stress, cell and tissue specificity, and the role of these gene products in modulating the IGF-1-AKT-mTOR pathways. Cancer Res..

[15] Kenzelmann Broz D (2013). Global genomic profiling reveals an extensive p53-regulated autophagy program contributing to key p53 responses. Genes Dev..

[16] Crighton D (2006). DRAM, a p53-induced modulator of autophagy, is critical for apoptosis. Cell.

[17] Scherz-Shouval R (2010). p53-dependent regulation of autophagy protein LC3 supports cancer cell survival under prolonged starvation. PNAS.

[18] Fridman JS, Lowe S (2003). Control of apoptosis by p53. Oncogene.

[19] Vaseva AV, Moll U (2009). The mitochondrial p53 pathway. Biochim. Biophys. Acta..

[20] Marchenko ND, Zaika A, Moll U (2000). Death signal-induced localization of p53 protein to mitochondria: A potential role in apoptotic signaling. J. Biol. Chem..

[21] Vainshtein A, Kazak L, Hood DA (2011). Effects of endurance training on apoptotic susceptibility in striated muscle. J. Appl. Physiol..

[22] Siu PM, Bryner RW, Martyn JK, Alway S (2004). Apoptotic adaptations from exercise training in skeletal and cardiac muscles. FASEB J..

[23] Adhihetty PJ, Ljubicic V, Hood DA (2007). Effect of chronic contractile activity on SS and IMF mitochondrial apoptotic susceptibility in skeletal muscle. Am. J. Physiol. Endocrinol. Metab..

[24] Stocks B, Dent JR, Joanisse S, McCurdy CE, Philp A (2017). Skeletal muscle fibre-specific knockout of p53 does not reduce mitochondrial content or enzyme activity. Front. Physiol..

[25] Fox DK (2014). p53 and ATF4 mediate distinct and additive pathways to skeletal muscle atrophy during limb immobilization. AJP Endocrinol. Metab..

[26] Saleem A, Iqbal S, Zhang Y, Hood DA (2015). Effect of p53 on mitochondrial morphology, import, and assembly in skeletal muscle. Am. J. Physiol.Cell Physiol..

[27] Safdar A (2016). Exercise-induced mitochondrial p53 repairs mtDNA mutations in mutator mice. Skelet. Muscle.

[28] Tachtsis B, Smiles WJ, Lane SC, Hawley JA, Camera D (2016). Acute endurance exercise induces nuclear p53 abundance in human skeletal muscle. Front. Physiol..

[29] Granata C, Oliveira RSF, Little JP, Renner K, Bishop D (2017). Sprint-interval but not continuous exercise increases PGC-1α protein content and p53 phosphorylation in nuclear fractions of human skeletal muscle. Sci. Rep..

[30] Zhuang J (2016). Forkhead box O3A (FOXO3) and the mitochondrial disulfide relay carrier (CHCHD4) regulate p53 protein nuclear activity in response to exercise. J. Biol. Chem..

[31] Bartlett JD (2013). Reduced carbohydrate availability enhances exercise-induced p53 signaling in human skeletal muscle: implications for mitochondrial biogenesis. AJP Regul. Integr. Comp. Physiol..

[32] Ljubicic V, Hood DA (2009). Specific attenuation of protein kinase phosphorylation in muscle with a high mitochondrial content. Am. J. Physiol. Endocrinol. Metab..

[33] Bartlett JD (2012). Matched work high-intensity interval and continuous running induce similar increases in PGC-1a mRNA, AMPK, p38, and p53 phosphorylation in human skeletal muscle. J. Appl. Physiol..

[34] Shieh SY, Ikeda M, Taya Y, Prives C (1997). DNA damage-induced phosphorylation of p53 alleviates inhibition by MDM2. Cell.

[35] Liang SH, Clarke M (2001). Regulation of p53 localization. Eur. J. Biochem..

[36] Irrcher I, Ljubicic V, Kirwan A, Hood DA (2008). AMP-activated protein kinase-regulated activation of the PGC-1α promoter in skeletal muscle cells. PLoS One.

[37] Lohrum MA, Woods DB, Ludwig RL, Bálint E, Vousden K (2001). C-terminal ubiquitination of p53 contributes to nuclear export. Mol. Cell. Biol..

[38] Marchenko ND, Wolff S, Erster S, Becker K, Moll U (2007). Monoubiquitylation promotes mitochondrial p53 translocation. EMBO J..

[39] Heyne K (2004). Identification of a putative p53 binding sequence within the human mitochondrial genome. FEBS Lett..

[40] Macleod KF (1995). p53-dependent and independent expression of p21 during cell growth, differentiation, and DNA damage. Genes Dev..

[41] Chipuk JE (2004). Direct activation of Bax by p53 mediates mitochondrial membrane permeabilization and apoptosis. Science.

[42] Matoba S (2006). p53 regulates mitochondrial respiration. Science.

[43] Chipuk JE, Green D (2006). Dissecting p53-dependent apoptosis. Cell Death Differ..

[44] Yu X (2013). Inhibition of autophagy via p53-mediated disruption of ULK1 in a SCA7 polyglutamine disease model. J. Mol. Neurosci..

[45] Kim Y, Hood DA (2017). Regulation of the autophagy system during chronic contractile activity-induced muscle adaptations. Physiol. Rep..

[46] Carter Heather N., Pauly Marion, Tryon Liam D., Hood David A. (2018). Effect of contractile activity on PGC-1α transcription in young and aged skeletal muscle. Journal of Applied Physiology.

[47] Chen CCW, Erlich AT, Hood DA (2018). Role of Parkin and endurance training on mitochondrial turnover in skeletal muscle. Skelet. Muscle.

[48] Cogswell AM, Stevens RJ, Hood DA (1993). Properties of skeletal muscle mitochondria isolated from subsarcolemmal and intermyofibrillar regions. Am. J. Physiol..

[49] Cooperstein SJ, Lazarow A (1951). A microspectrophotometric method for the determination of cytochrome oxidase. J. Biol. Chem..

