# Supplementary material for: The Role of p53 in Determining Mitochondrial Adaptations to Endurance Training in Skeletal Muscle
Source: Sci Rep. 2018 Oct 2;8:14710. doi: 10.1038/s41598-018-32887-0 (PMC6168598; doi:10.1038/s41598-018-32887-0)
Supplement: Supplementary file 1 — Supplementary Information [file 41598_2018_32887_MOESM1_ESM.docx]

**Supplemental Information**

**The Role of p53 in Determining Mitochondrial Adaptations to Endurance Training in Skeletal Muscle**

Kaitlyn Beyfuss, Avigail T. Erlich, Matthew Triolo, David A. Hood*

Muscle Health Research Centre, School of Kinesiology and Health Science, York University, Toronto, Ontario, M3J 1P3, Canada

*Corresponding Author: Dr. David Hood; School of Kinesiology and Health Science, York University, Toronto, ON, Canada M3J 1P3; (Email) dhood@yorku.ca.

**Supplemental Methods**

***Animal Genotyping.*** Progeny of breeding pairs were genotyped using ear clippings obtained from each mouse for crude DNA extraction. Extracts were then added to a polymerase chain reaction (PCR) tube containing DNA Taq polymerase (Sigma Jumpstart REDtaq Ready Mix PCR Reaction Mix). Forward and reverse primers for the WT and KO *p53* gene were added to test the genotype of the whole body mice, whereas for the muscle-specific mice, a forward and reverse primer for the *Cre* gene was added. Genomic differences were detected using PCR amplification and the reaction products were separated on 2% agarose gels at 120 V for ~one hour and visualized with the ethidium bromide.

***Experimental Design.*** All mice were acclimatized to the treadmill for two days prior to the first graded exercise performance test. Acclimatization on day one involved a 5 min resting bout on the treadmill to familiarize, followed by a short exercise bout at 5 m/min for 5 min. The second day involved a similar 5 min resting bout, followed by exercise at 5 m/min for 5 min and 10 m/min for 10 min. After 24 hours, mice underwent the first exhaustive exercise performance test. Animals commenced at 5m/min for 5 min, followed by 10 m/min for 10 min, then increasing from 15 m/min to 20 to 25 m/min for 5 min each before beginning the exhaustive portion of increased the speed by 1 m/min every 3 min until exhaustion. Lactate measurements were obtained prior to the test and following removal from the treadmill to ensure that exhaustion was reached. Mice were then randomized to a sedentary or training group. The sedentary group involved no treadmill exercise for 6 weeks, while the training group participated in a 6-week training protocol exercising 5 days/week and beginning at 5 m/min for 20 min and progressing to 26 m/min for 90 min. Succeeding the 6 weeks, both the sedentary and training groups underwent a second exhaustive exercise performance test, 48 hours following the training program. Approximately 48 hours later, all mice underwent an acute bout of treadmill exercise at 15 m/min for 90 min. All mice were sacrificed by cervical dislocation immediately following the acute bout for instantaneous tissue removal of skeletal muscle, cardiac tissue, and epididymal fat. See supplemental Figure 1 for an outline of the exercise protocol.

***Protein Concentration.*** The Bradford protein assay was used to determine the protein concentration of samples. Briefly, to standardize for concentration, bovine serum albumin (2mg/ml) was combined with double distilled water and Sakamoto extraction buffer in eppendorf tubes. Protein extracts were mixed with double- distilled water and analyzed in comparison with the standard curve using a Bio-Tek Synergy HT micro plate reader.

***Mitochondrial Respiration.*** Isolated SS and IMF mitochondria (50 μl) were incubated with 250 μl of V̇o_2_ buffer (in mM: 250 sucrose, 50 KCl, 25 Tris·HCl, 10 K_2_HPO_4_, pH=7.4), at 30°C in a water-jacketed respiratory chamber with continuous stirring. Respiration rates (nanoatoms O_2_·min^−1^·mg^−1^) were evaluated in the presence of 10 mM glutamate (state 4 or passive respiration) and 0.44 mM ADP (state 3 or active respiration) with the use of a Clark oxygen electrode.

***Mitochondrial Reactive Oxygen Species (ROS) Production.*** SS and IMF mitochondria (50 μg) were incubated with 50 μM dichlorodihydro-fluorescein diacetate (H_2_DCF-DA) and V̇o_2_ buffer at 37°C for 30 min in a polystyrene 96-well plate. The fluorescence emission (between 485 and 528 nm) is directly proportional to ROS production and was measured with a Synergy HT microplate reader. ROS production was assessed during state 4 and state 3 respiration by the addition of 10 mM glutamate and 0.44 mM ADP respectively, to isolated mitochondria immediately before the addition of H_2_DCF-DA.

***Protein Release Assay.*** Isolated SS and IMF mitochondrial fractions (150 μg) were incubated in resuspension medium for 60 min at 30°C. Reaction mixtures were subsequently centrifuged at 14,000 g (4°C) to pellet mitochondria, and the supernate was analyzed for cytochrome c and AIF release from the mitochondria by Western blot analysis. To ensure accurate comparison of bands on different blots, all blots were exposed for the same amount of time.

***mRNA Expression Analyses.*** The mRNA expression of *SCO2, TIGAR, Mdm2, p62, LC3, p52, p21, Bax, PGC-1α*, and *Tfam* were quantified using the 7500 Real-Time PCR system (Applied Biosystems Inc., Foster City, CA, USA) and SYBR® Green chemistry (PerfeC_T_a SYBR® Green Supermix, ROX, Quanta BioSciences, Gaithersburg, MD, USA). First-strand cDNA synthesis from 2µg of total RNA was performed with primers using Superscript III transcriptase (Invitrogen) according to the manufacturer’s directions. Forward and reverse primers (Table 2) for the aforementioned genes were designed based on sequences available in GenBank (http://www.ncbi.nlm.nih.gov/entrez/query.fcgi) using the MIT Primer 3 designer software (http://wi.mit.edu/cgi-bin/primer3/primer3_www.cgi), and were confirmed for specificity using the basic local alignment search tool (www.ncbi.nlm.nih.gov/BLAST/). *B2M* and *GAPDH* were used as housekeeping genes, the expression of which did not change between conditions, genotype and rodent model. Each well within a 96-well plate contained: SYBR ® Green SuperMix, forward and reverse primers (20 µM), sterile H_2_O and 10 ng of cDNA, for a final reaction volume of 25 µl. All samples were run in duplicate simultaneously with negative controls that contained no cDNA. The PCR program consisted of an initial holding stage (95°C for 10 min), an amplification phase (40 cycles at 60°C for 1 min, 95°C for 15 sec), and melting stage (95°C for 15 sec, 60°C for 1 min, 95°C for 25 sec). Melting point dissociation curves generated by the instrument were used to confirm the specificity of the amplified product. Primer efficiency curves were generated for each set to ensure 100 ± 2% efficiency. For quantification, the threshold cycle (CT) number of endogenous references genes was subtracted from the CT number of the target gene [ΔCT = CT(target) – CT(reference)] . The ΔCT value of the control tissue was subtracted from the ΔCT value of the experimental tissue [ΔΔCT = ΔCT (experimental) - ΔCT(control}]. Results were reported as fold-changes using the ΔΔCT, calculated as 2^-ΔΔCT^.

**Supplemental Figures**

**Figure S1. Exercise training protocol.**


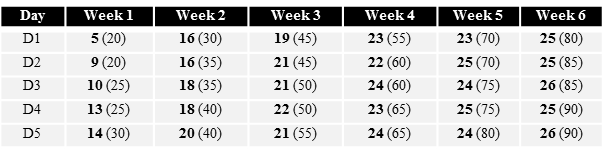

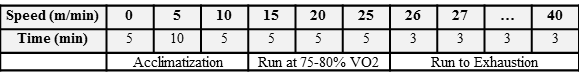


**6-week Chronic Endurance Exercise Program**

**Acute Exercise Bout to induce gene expression**

**Immediate Tissue Extraction**

**Post-Training Period: Exercise Performance Stress Test**

**Pre-Training Exhaustive Stress Test**

**6-week Sedentary**

48 hrs

**Two-day Treadmill Acclimatization**

Muscle specific (MS) WT and p53 mKO mice and whole body (WB) WT and p53 KO mice underwent a two-day acclimatization program to the treadmill, followed by an exhaustive performance test to measure baseline differences between mouse model and genotype. Mice were randomized to a training and sedentary arm. Mice in the training group underwent a 6-week progressive training program. Speed was expressed in m/min and (time) was measured in minutes. Once individual programs were completed, all mice underwent a second exhaustive performance test to measure training adaptations. Following 48 hours, animals were subjected to an acute bout of exercise to upregulate transcriptional signaling, followed by immediate extraction of the skeletal muscles, heart, and epididymal fat.

**Figure S2. Antioxidant regulation with exercise training.**

**
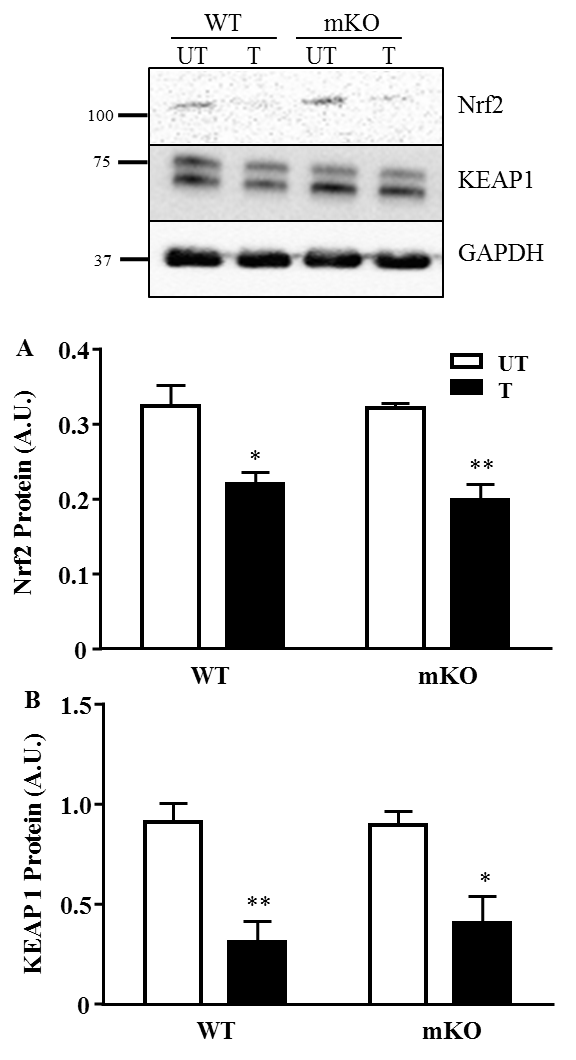
**

**A)** The major antioxidant transcriptional regulator Nrf2, and its **B)** negative regulator KEAP1 were examined in the context of exercise training in mKO mice (n=4-5/group); *p≤0.05, **p≤0.05, UT vs. T, 2-way ANOVA. Exercise training reduced the antioxidant transcriptional activator and its negative regulator. No difference in antioxidant regulation occurred in the absence of p53. Data are presented as mean ± SEM.


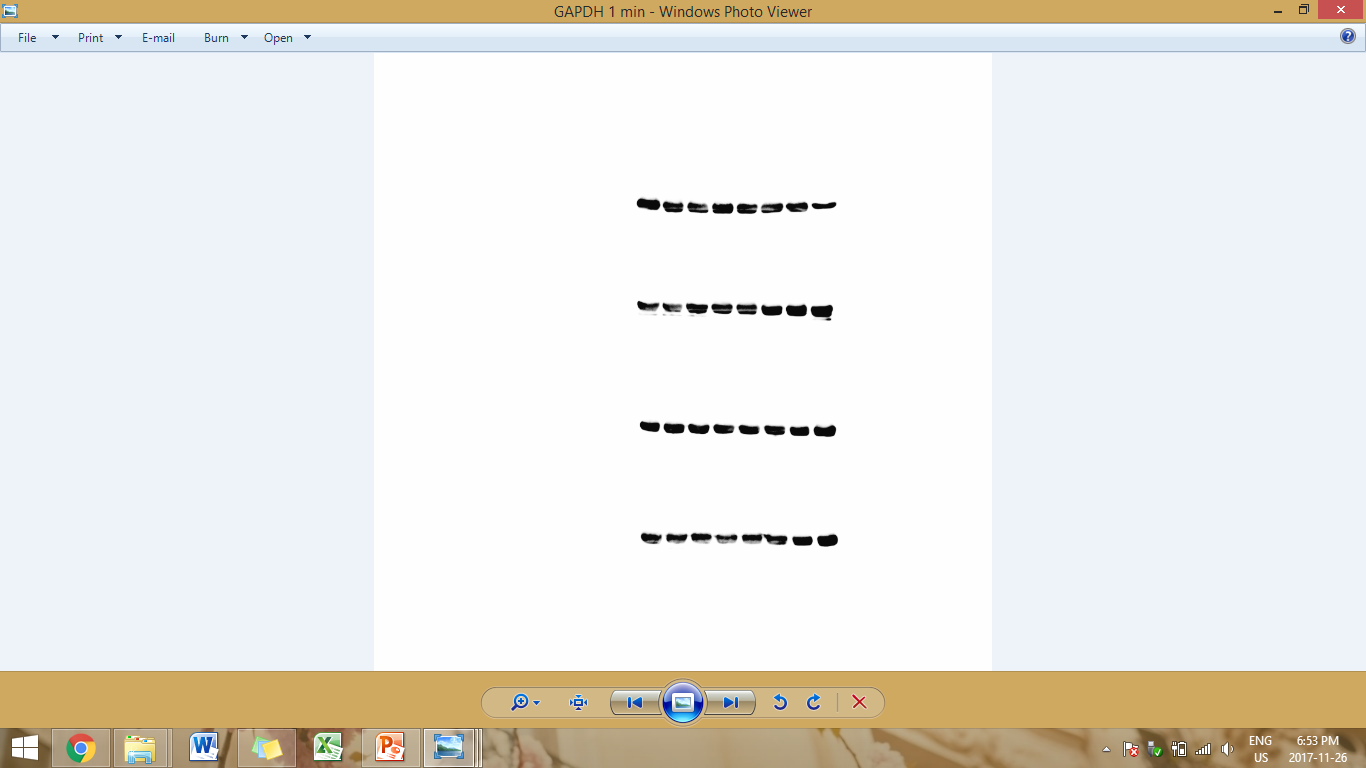

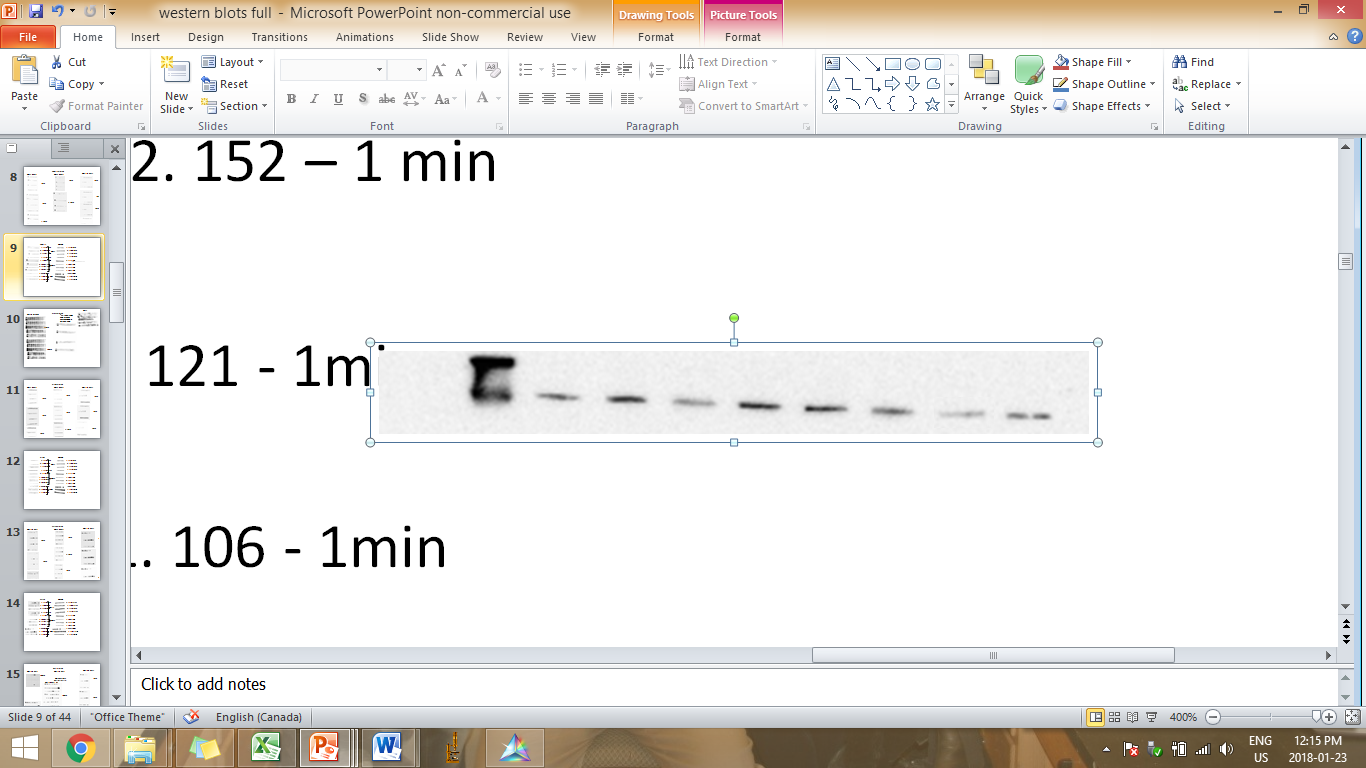


PGC-1α

GAPDH

UT T UT T UT T UT T

WT mKO WT KO

Muscle Specific Whole Body

100

37

**Figure S3. Full blot images for Fig. 6C**

C T C T C T C T

SS IMF SS IMF

WT UT WT T


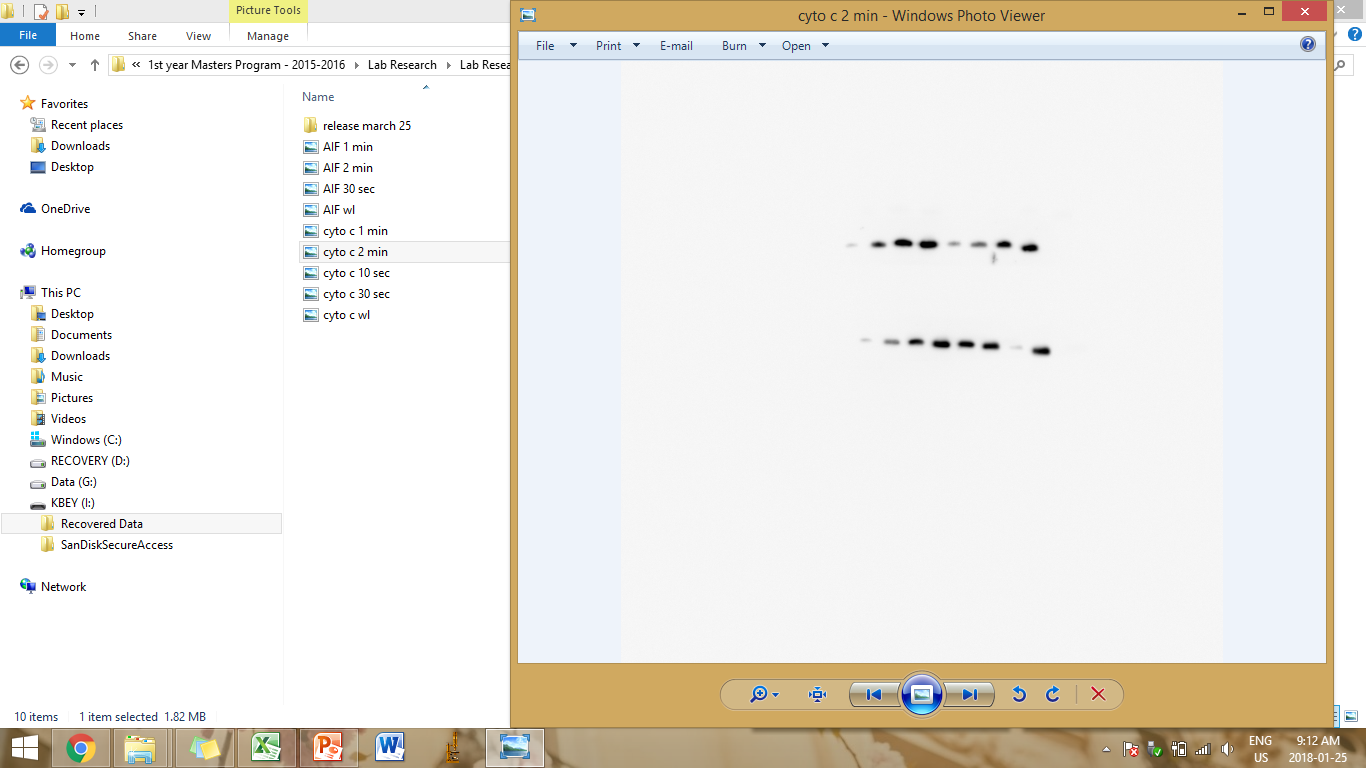

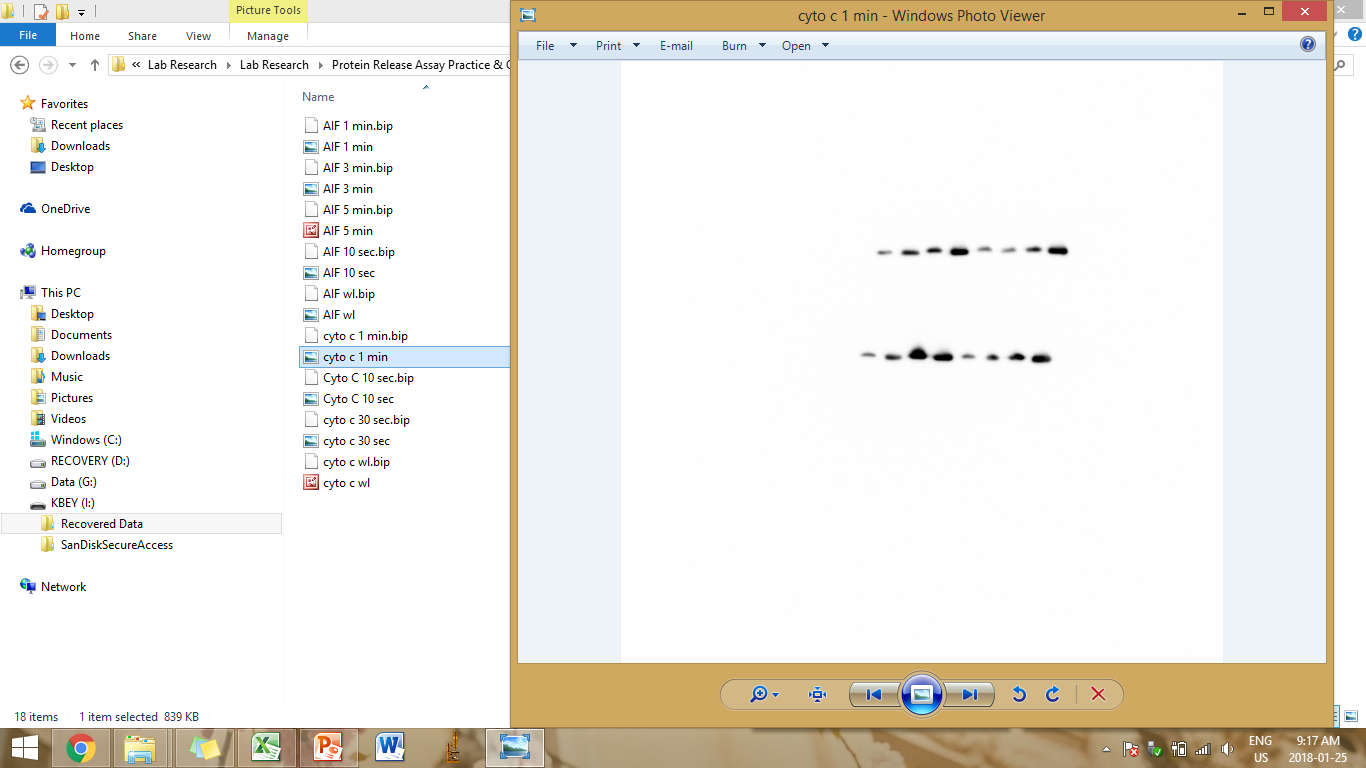


C T C T C T C T

SS IMF SS IMF

mKO UT mKO T

15

Cytochrome c

Cytochrome c

15

Muscle Specific Mice

C T C T C T C T

SS IMF SS IMF

WT UT WT T

C T C T C T C T

SS IMF SS IMF

KO UT KO T


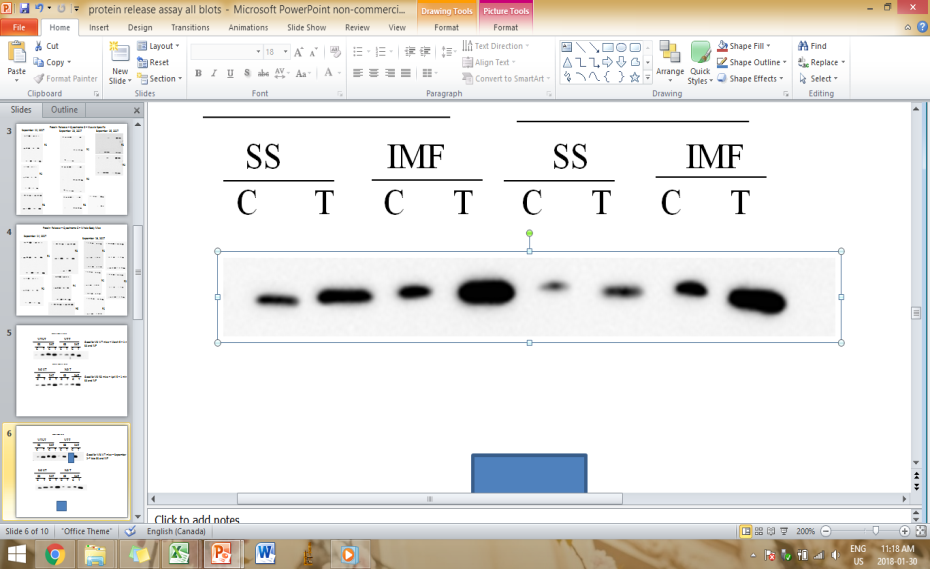

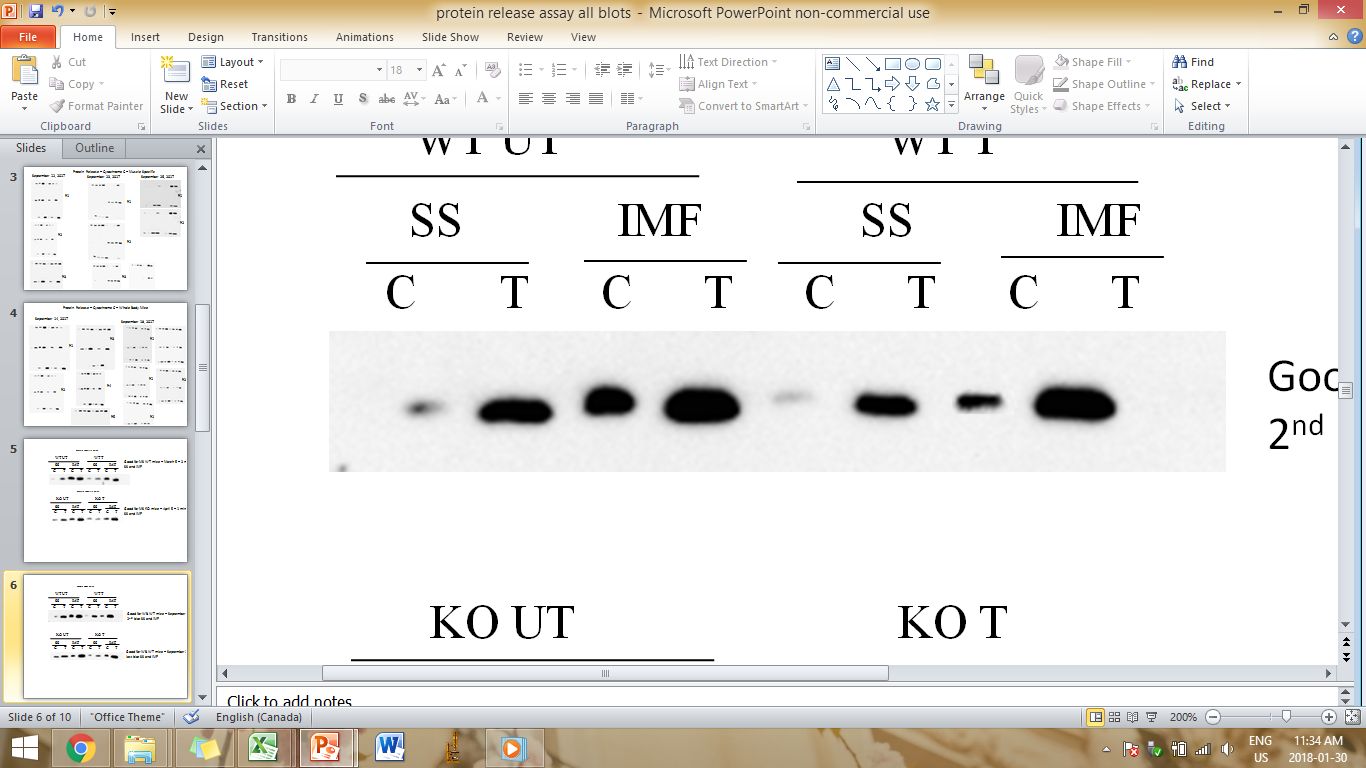


Whole Body Mice


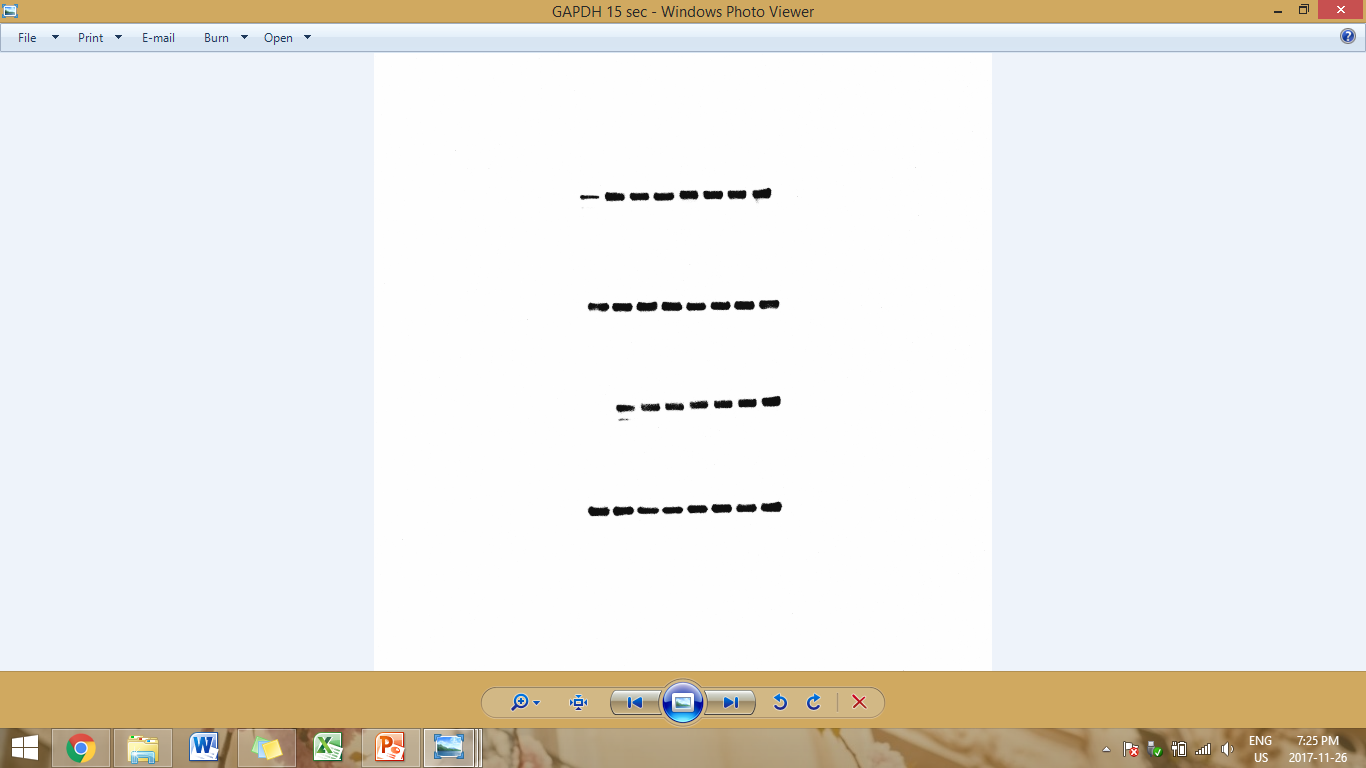

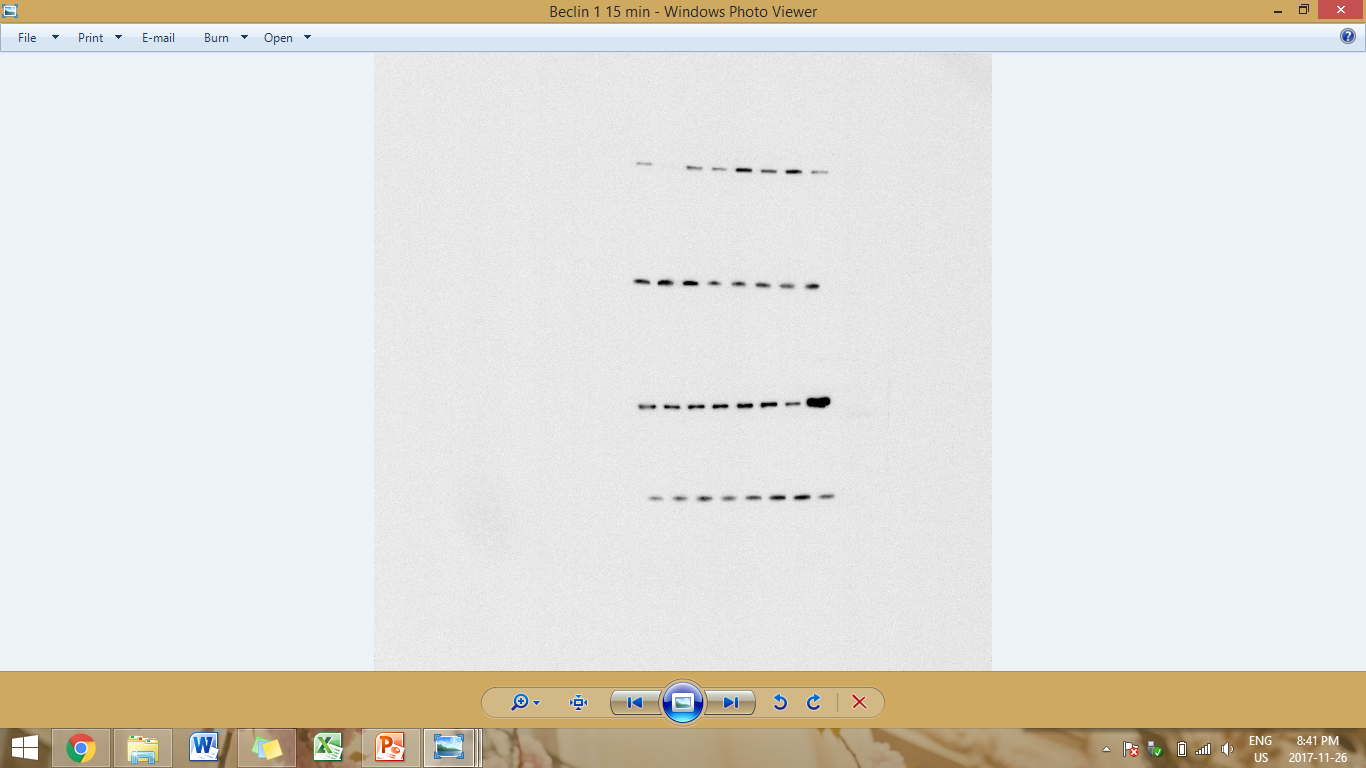

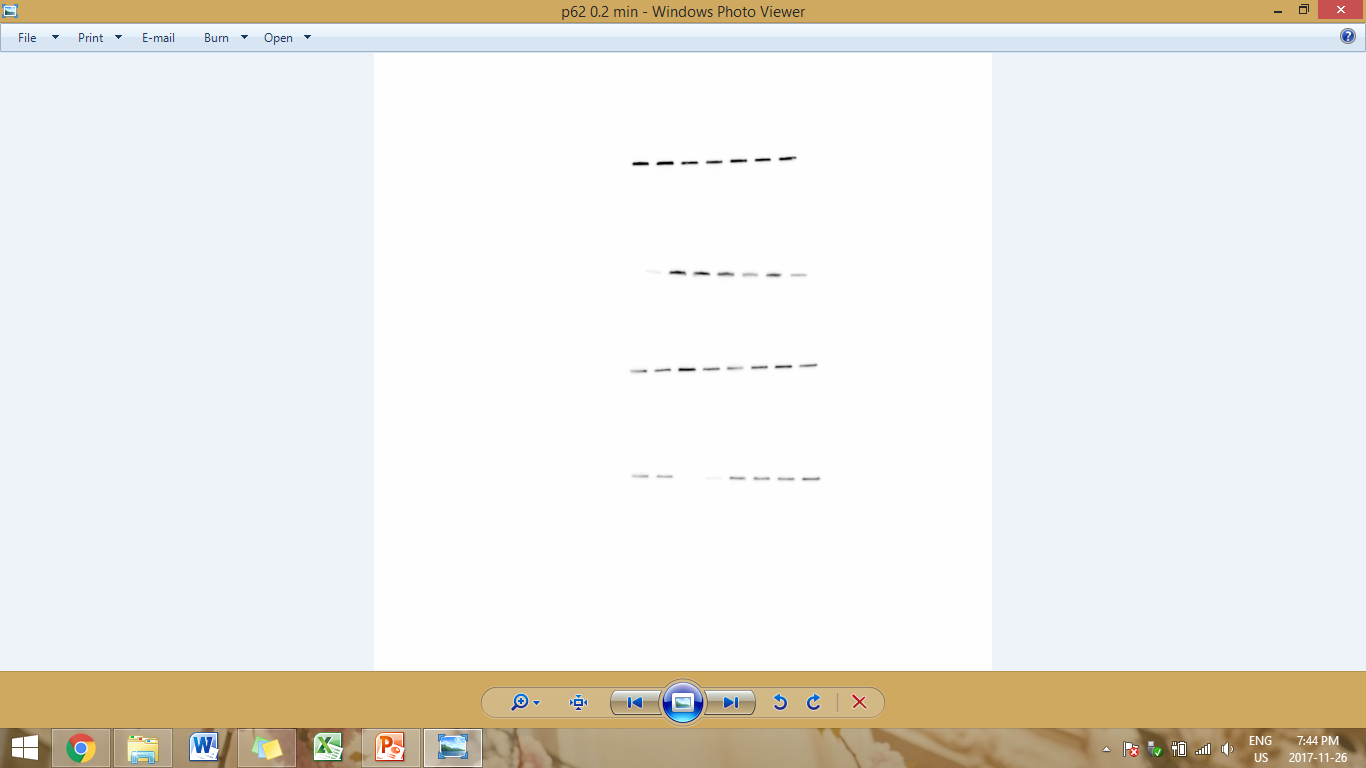

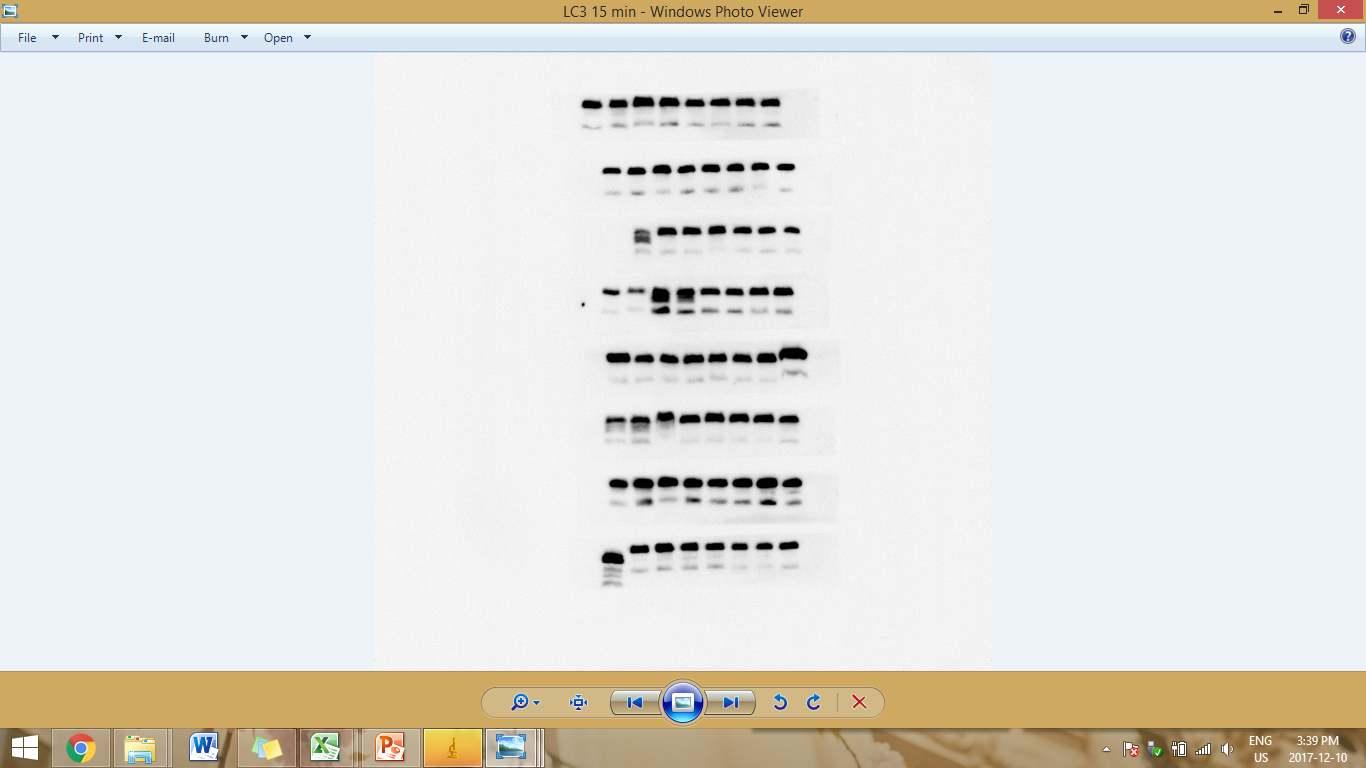


LC3

p62

GAPDH

UT T UT T UT T UT T

WT mKO WT KO

Muscle Specific Whole Body

15

75

50

Beclin-1

37

**Figure S4. Full blot images for Fig. 6F**

**Fig. S5. Full blot images for Fig. 6G**

**Figure S6. Skeletal muscle performance and neurological function in the absence of p53.**


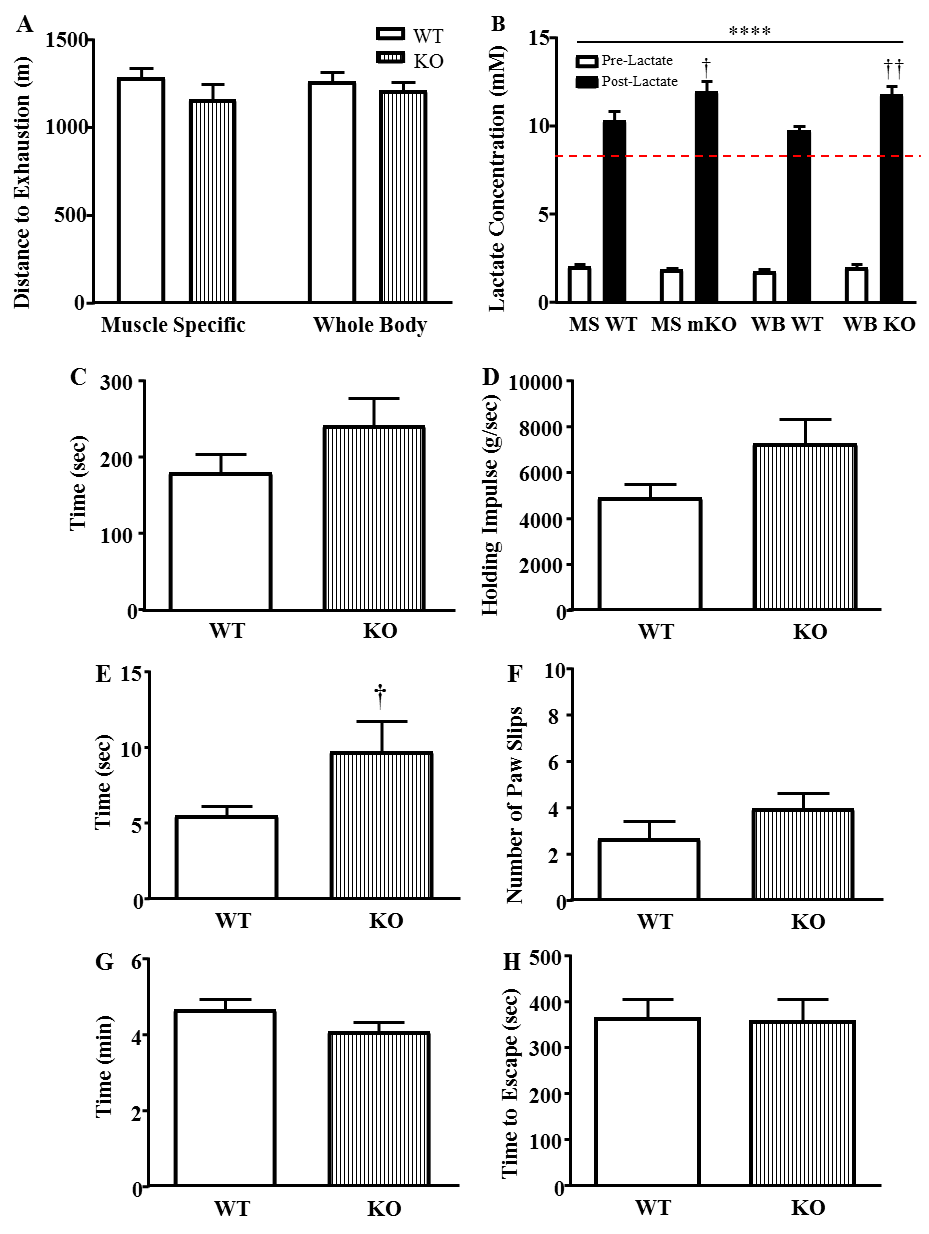


mKO and WB mice were subjected to a pre-training exhaustive bout of exercise. Exercise performance was measured by recording A) distance to exhaustion (n=17-18/group); N.S. p>0.05, 2-way ANOVA, and B) lactate production pre- and post-exercise (n=7-20/group); †p≤0.05, ††p≤0.01, WT vs. KO, 2-way ANOVA; ****p≤0.001, Pre-lactate vs. Post-lactate (effect of exercise), 2-way ANOVA. The red line indicates an exhaustive lactate threshold (>8mM). To determine if any deficit in skeletal muscle strength exists as a result of the whole-body p53 deletion, a cage hanging test was performed. Strength was measured by **C)** length of time spent hanging (n=14/group). N.S., p>0.05, WT vs KO, student’s T-test, and by the **D)** holding impulse calculated according to Holding Impulse = Body Mass (g) x Time Spent Hanging (sec) (n=14/group). N.S., p>0.05, WT vs KO, student’s T-test. Data are presented as mean ± SEM. This model for resistance performance indicated that KO mice, though not significantly different from the WT mice, did display an increased trend for enhanced strength capacity. As no significant difference in aerobic and anaerobic exercise performance was determined, neurological testing was used to determine if functional deficits may occur as a result of the whole-body absence of p53. To examine walking synchronization in the WB mice, a balance beam test was employed to measure the **E)** time to cross (n=14/group); †p≤0.05, WT vs. KO, Student’s t-test, and the **F)** number of paw slips (n=14/group); N.S., p>0.05, Student’s t-test. A pole test was employed to examine motor coordination and synchronization for correct paw placement during vertical navigation down a pole by measuring **G)** the time to traverse down the pole (n=14/group); N.S., p>0.05, Student’s test. To establish central neural activity for voluntary movement identified through spatial learning, memory, and behavioural parameters, a cylinder escape test was employed. **H)** The time to escape the cylinder was measured (n=14/group); N.S., p>0.05. Neurological testing revealed delayed time to crossing the beam indicative of impaired walking synchronization, however the other tests did not reveal impairments in central neural activity. Data are presented as mean ± SEM.

**Figure S7. Apoptotic signaling under basal and exercise training conditions.**


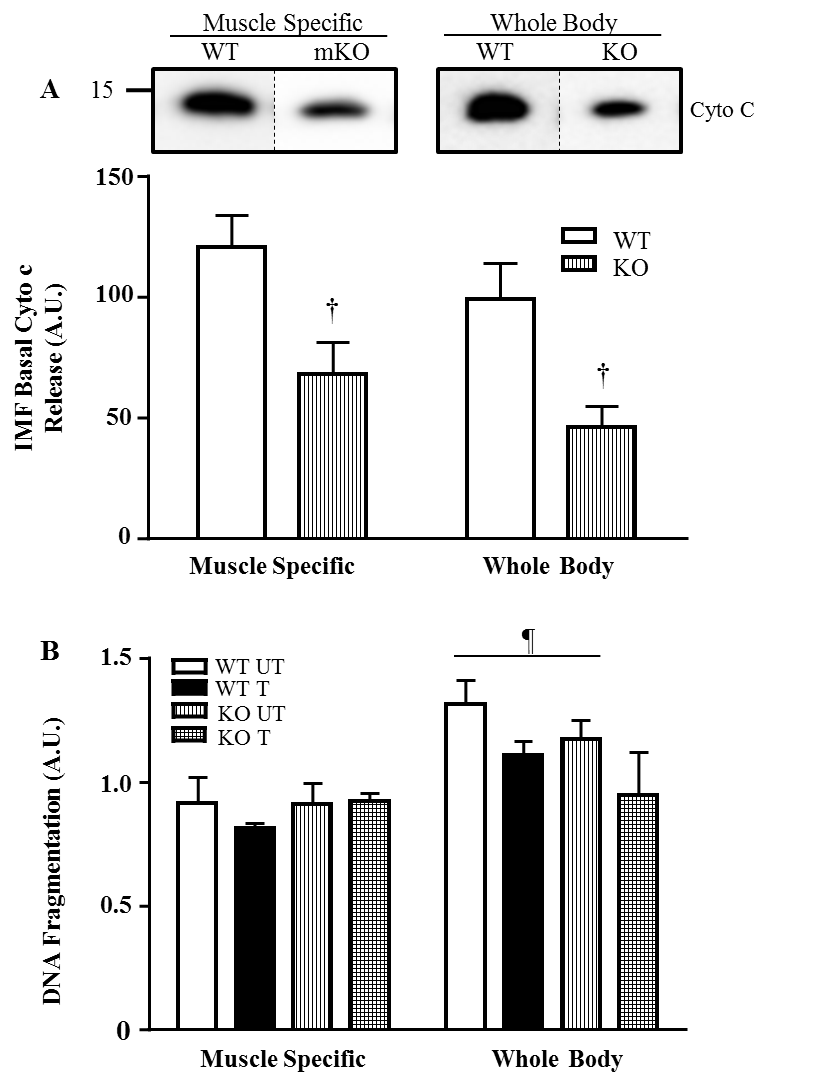


**A)** Apoptosis was basally measured through cytochrome c release from IMF mitochondria (n=6-14/group); †p≤0.05, UT WT vs, KO, Student’s t-test. In both the mKO and WB mice in the absence of p53, there is a decrease in cytochrome c release, indicative of reduced mitochondrial-induced apoptosis. **B)** DNA fragmentation, a consequence following the release of mitochondrial proteins, was measured following training in both mouse models. Though no significant difference was detected with the effect of training and genotype, the WB mice do experience greater apoptosis than the mKO mice (n=6-8/group); ¶ p≤0.05, WB vs. MS, Student’s t-test. Furthermore, a trend for reduced DNA fragmentation with training in the WB WT and KO mice was observed. Data are presented as mean ± SEM.

**Figure S8. Signaling regulation in untrained and trained mKO and WB mice.**

**
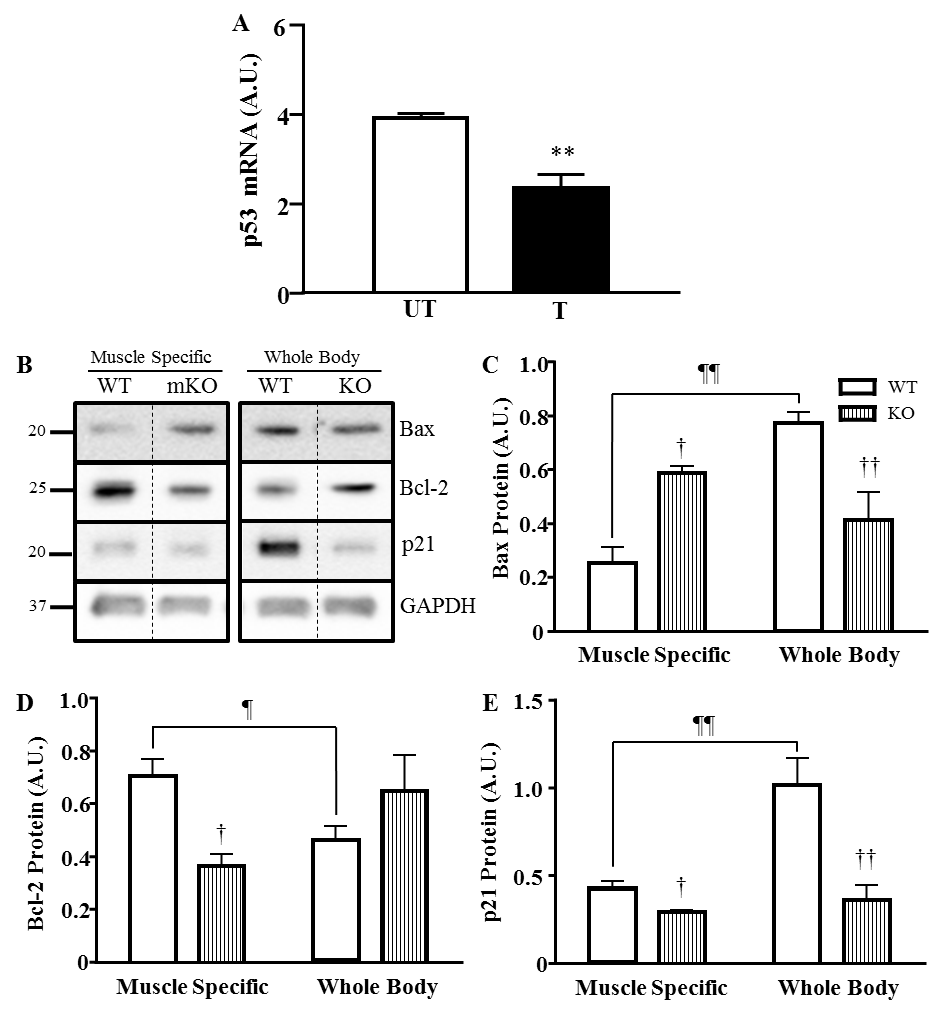
**

**A)** p53 mRNA was reduced with training in mKO mice (n=6-10/group); **p≤0.01, UT vs. T, Student’s t-test. **B)** The effect of the absence of p53 on apoptotic signaling in untrained mKO and WB mice was measured through protein analysis of **C)** Bax (n=4-6/group); †p≤0.05; ††p≤0.01, UT WT vs. KO; ¶¶p≤0.01, mKO vs. WB, 2-way ANOVA, **D)** Bcl-2 (n=5-8/group); †p≤0.05, UT WT vs. KO, 2-way ANOVA; ¶p≤0.05, mKO vs. WB, Student’s t-test, and **E)** p21 (n=6-8/group); †p≤0.05, ††p≤0.01, UT WT vs. KO, Student’s t-test and 2-way ANOVA; ¶¶p≤0.01, mKO vs. WB, 2-way ANOVA. Differential regulation of apoptotic and cellular senescent protein levels occurs in the absence of p53 basally between the two mouse models. Data are presented as mean ± SEM.

**Supplemental Tables**

**Table S1. List of primary and secondary antibodies for immunoblotting.**

| **Antibody** | **Product Number** | **Company** |
| --- | --- | --- |
| **Upstream Regulators of p53** | | |
| Mdm2 | n/a | Provided by Dr. Olivier Birot |
| CHCHD4 | Sc-98628 | Santa Cruz Biotechnology |
| Phospho-p53 (ser15) | 9284S | Cell Signaling |
| Total-p53 | PAB 421 | Provided by Dr. Samuel Benchimol |
| **Autophagy Markers** | | |
| LC3 A/B | 4108 S | Cell Signaling |
| p62 | Ab56416 | Abcam |
| Parkin | 4211 S | Cell Signaling |
| Beclin 1 | 3738 S | Cell Signaling |
| **Antioxidant Markers** | | |
| KEAP1 | 10503-2-AP | Proteintech |
| Nrf2 | Sc-722 | Santa Cruz Biotechnology |
| **Mitochondrial Biogenesis Markers** | | |
| PGC-1α | ab3242 | EMD Millipore |
| Tfam | n/a | Antibody made in house |
| COX IV | ab14744 | Abcam |
| **Cellular Senescent and Apoptosis Markers** | | |
| p21 | Sc-397 | Santa Cruz Biotechnology |
| Bcl-2 | Sc-7382 | Santa Cruz Biotechnology |
| Bax | Sc-493 | Santa Cruz Biotechnology |
| Cytochrome C | 556433 | BD Pharmingen |
| **Loading Controls** | | |
| GAPDH | Ab8245 | Abcam |
| α-Tubulin | CP06-100ug | Calbiochem |
| VDAC | ab14734 | Abcam |
| H2B | 2934 S | Cell Signaling |

**Table S2. List of primer oligonucleotide sequences used in real-time qPCR analysis for *Mus Musculus.***

| Gene | Forward Primer (5' 🡪 3') | Reverse Primer (5' 🡪 3') |
| --- | --- | --- |
| *PGC-1a* | TTCCACCAAGAGCAAGTAT | CGCTGTCCCATGAGGTATT |
| *Tfam* | GAAGGGAATGGGAAAGGTAGA | AACAGGACATGGAAAGCAGAT |
| *p53* | CCGACCTATCCTTACCATCATC | TTCTTCTGTACGGCGGTCTC |
| *Mdm2* | TCAGACAGGAGAAAGCGATACA | CACGAAGGGTCCAGCATCTT |
| *SCO2* | TCCCTTCACCCTTCGCTGAAC | CAGTAGCATCGTGGACCTGAA |
| *TIGAR* | CATTCAAGGACAAGGCGTAGAT | TGGAGAAGGCGTGGGTAAA |
| *p21* | CACCACCAAGCCATTCCATA | ACTGCCAATCACCACACTAT |
| *p62* | TGTGGTGGGAACTCGCTATAA | CAGCGGCTATGAGAGAAGCTA |
| *LC3* | GCTTGCAGCTCAATGCTAAC | CCTGCGAGGCATAAACCATGT |
| *GAPDH* | AACACTGAGCATCTCCCTCA | GTGGGTGCAGCGAACTTTAT |
| *b2 microglobulin* | GGTCTTTCTGGTGCTTGTCT | TATGTTCGGCTTCCCATTCT |

PGC-1α, peroxisome proliferator-activated receptor-γ coactivator-1 α; Tfam, mitochondrial transcription factor; Mdm2, Mouse double minute 2 homolog; SCO2, synthesis of cytochrome-*c* oxidase 2; TIGAR, TP53-inducible glycolysis and apoptosis regulator; LC3, light chain 3; GAPDH, Glyceraldehyde 3-phosphate dehydrogenase; *b2 microglobulin*, beta-2 microglobulin.
